# Transcriptionally induced nucleoid-associated protein-like *ccr1* in combined-culture serves as a global effector of *Streptomyces* secondary metabolism

**DOI:** 10.3389/fmicb.2024.1422977

**Published:** 2024-07-12

**Authors:** Yukun Lei, Hiroyasu Onaka, Shumpei Asamizu

**Affiliations:** ^1^Graduate School of Agricultural and Life Sciences, The University of Tokyo, Tokyo, Japan; ^2^Collaborative Research Institute for Innovative Microbiology, The University of Tokyo, Tokyo, Japan; ^3^Department of Life Science, Faculty of Science, Gakushuin University, Tokyo, Japan; ^4^Engineering Biology Research Center, Kobe University, Kobe, Japan

**Keywords:** *Streptomyces*, secondary metabolism, co-culture, bacterial interaction, regulation, nucleoid-associated protein

## Abstract

Combined-cultures involving mycolic acid-containing bacteria (MACB) can stimulate secondary metabolite (SM) production in actinomycetes. In a prior investigation, we screened *Streptomyces coelicolor* JCM4020 mutants with diminished production of SMs, specifically undecylprodigiosin (RED), which was enhanced by introducing the MACB *Tsukamurella pulmonis* TP-B0596. In this study, we conducted mutational analysis that pinpointed the *sco1842* gene, which we assigned the gene name *ccr1* (combined-culture related regulatory protein no. 1), as a crucial factor in the deficient phenotype observed in the production of various major SMs in *S. coelicolor* A3(2). Notably, the Ccr1 (SCO1842) homolog was found to be highly conserved throughout the *Streptomyces* genome. Although Ccr1 lacked conserved motifs, in-depth examination revealed the presence of a helix–turn–helix (HTH) motif in the N-terminal region and a helicase C-terminal domain (HCTD) motif in the C-terminal region in some of its homologs. Ccr1 was predicted to be a nucleoid-associated protein (NAP), and its impact on gene transcription was validated by RNA-seq analysis that revealed genome-wide variations. Furthermore, RT-qPCR demonstrated that *ccr1* was transcriptionally activated in combined-culture with *T. pulmonis*, which indicated that Ccr1 is involved in the response to bacterial interaction. We then investigated *Streptomyces nigrescens* HEK616 in combined-culture, and the knockout mutant of the *ccr1* homolog displayed reduced production of streptoaminals and 5aTHQs. This finding reveals that the Ccr1 homolog in *Streptomyces* species is associated with SM production. Our study elucidates the existence of a new family of NAP-like proteins that evolved in *Streptomyces* species and play a pivotal role in SM production.

## 1 Introduction

*Streptomyces* species are ubiquitous soil bacteria well-known for producing antimicrobial secondary metabolites (SMs) (Barka et al., 2016; Parra et al., 2023). Analysis of numerous *Streptomyces* genome sequences has revealed that they harbor a wide array of biosynthetic gene clusters (BGCs) responsible for SM production, and BGCs have significant, untapped potential for elucidating SM biosynthesis (Gavriilidou et al., 2022). Although only a limited number of BGCs have been successfully expressed under laboratory culture conditions, and most of these regions remain unexplored, various strategies have been devised to identify the metabolites encoded by these hidden BGCs (Zarins-Tutt et al., 2016; Lee et al., 2021). The presence of numerous diverse and conserved BGCs in these soil bacteria may confer advantages for their survival in natural environments, including in competition for nutritional resources with other microorganisms (Van der Meij et al., 2017). For example, SM production by *Streptomyces* is a complex process closely intertwined with cell metabolism (Wang et al., 2020). Furthermore, some of these processes are intricately linked to and regulated by cell differentiation (van der Heul et al., 2018) and can be induced by environmental stresses such as cell envelope damage (Hesketh et al., 2011), oxidative stresses (Sulheim et al., 2020), and heat shock stresses (Lu et al., 2021; Saito et al., 2022).

Several cluster-situated and global regulators involved in SM production have been thoroughly characterized (van der Heul et al., 2018), but random mutagenesis experiments have revealed “orphan” (standalone) genes that seem to indirectly influence SM production (Gehring et al., 2000; Xu et al., 2017). Clarifying the role of these orphan genes in SM production presents challenges; nonetheless, deciphering these uncharacterized orphan systems, which impact SM production, is crucial for gaining deeper insight into how SM production systems are integrated into the overall cell system.

The combined-culture technique involves co-culturing actinomycetes with mycolic acid-containing bacteria (MACB); e.g., *Tsukamurella pulmonis* TP-B0596, which has been used to stimulate SM production in actinomycetes (Onaka et al., 2011; Asamizu et al., 2015; Kato et al., 2022). This method yielded significant success and resulted in the isolation of 44 new SMs from 15 different actinomycetes so far (Supplementary Table S1). In a previous study, we documented the creation of a mutant library of *S. coelicolor* JCM4020 using a carbon ion (^12^C^5+^) beam to screen for mutants displaying varying levels of undecylprodigiosin (RED) production (Yanagisawa et al., 2022). This approach was used to identify the genes responsible for RED production during interactions with *T. pulmonis*, which could contain a yet-unknown regulation system. Out of ~152,000 irradiated spores, we identified 86 mutants that exhibited a phenotype characterized by decreased RED production while maintaining apparent normal growth on minimal medium (Yanagisawa et al., 2022). We analyzed point mutations induced by carbon-ion beam irradiation in 16 randomly selected mutants and revealed that the inactivation of genes such as *gltB* (glutamate synthase, *sco2026*), *fusA* (elongation factor G, *sco4661*), and *sarA* (a hypothetical membrane protein, *sco4069*) led to reduced RED production (Yanagisawa et al., 2022). Additionally, further investigation of remaining mutants revealed that inactivation of *sco1718* (a TetR family transcriptional regulator, TFR) leads to reduced production of SMs, including RED, which is caused by overexpression of adjacent *sco1719-20* genes encoding ATP-binding cassette (ABC) transporters (Lei et al., 2023). Furthermore, we also found two other TFR-ABC transporter gene sets (*sco4358-4360* and *sco5384-5382*) in the genome that can affect SM production (Lei et al., 2023). In this study, we used a forward genetics approach to further investigate the acquired mutants and identified *ccr1* (*sco1842*) as the causative factor behind the observed reduction in RED production. We report the impact of *ccr1* on SM production in two *Streptomyces* species: *S. coelicolor* A3(2) and *S. nigrescens* HEK616.

## 2 Materials and methods

### 2.1 Bacterial strains and culture conditions

*S. coelicolor* JCM4020 was used for mutagenesis experiments as previously described (Yanagisawa et al., 2022; Lei et al., 2023). *S. coelicolor* A3(2) was used for gene knockout and phenotype analysis. *Streptomyces* strains were grown on MS agar medium (Kieser et al., 2000) for sporulation and on Bennett's glucose agar medium (Kieser et al., 2000) for general cultivation. MS agar with 10 mM MgCl_2_ was used for *Escherichia coli*-mediated conjugal transfer of DNA (Kieser et al., 2000). *E. coli* DH5α was used as the general cloning host, and *E. coli* ET12567 (pUZ8002) was used as the plasmid donor in intergeneric conjugation (Kieser et al., 2000). The *E. coli* strains were grown in Luria broth supplemented with antibiotics. Apramycin (50 μg/mL for *E. coli*, 25 μg/mL for *Streptomyces* species), chloramphenicol (25 μg/mL), kanamycin (50 μg/mL), and carbenicillin (100 μg/mL) were added to the growth media as required.

Media used for RED production in mono-culture and combined-culture included PGA [components (g/L): peptone, 5; glycerol, 10; and agar, 20 (pH 7.1)] (Yepes et al., 2011), R2YE medium (Kieser et al., 2000), and YGGS medium [components (g/L): yeast extract, 3; glucose, 10; glycerol, 20; and soluble starch, 20; (pH 7.2)] (Yanagisawa et al., 2022; Lei et al., 2023). Genomic DNA of actinomycetes was isolated using CTAB protocol (Kieser et al., 2000) and purified by DNeasy PowerClean Pro CleanUp Kit (Qiagen, Hilden, Germany) according to the manufacturer's protocol for next-generation sequencing. Complete genomic DNA sequences of *S. coelicolor* JCM4020 and *S. nigrescens* HEK616 were previously deposited in National Center for Biotechnology Information's GenBank (AP025454 and AP026073, respectively).

### 2.2 Genome re-sequencing

Genome re-sequencing of Mt-206005 was performed by Chemical Dojin (Kumamoto, Japan) using an Illumina NovaSeq 6000 System (Illumina, San Diego, CA, USA). The obtained Illumina short-read data in FASTA format were imported and analyzed using CLC Genomic Workbench software ver. 10 (Qiagen). After mapping the short reads to the reference genome sequences of JCM4020, nucleotide substitutions, insertions, and deletions were detected by comparison with the reference wild-type sequence data obtained at the same time. Large deletions of the genome were manually searched. The identified point mutations were confirmed by Sanger sequencing of the PCR-amplified products.

### 2.3 RNA-seq analysis

Parent strain A3(2) and knockout strain Δ*ccr1* were grown in a 79NG agar plate covered with cellophane membrane for 48 h, and the total RNA was isolated from the cells following previously published protocols (Asamizu et al., 2022a; Lei et al., 2023). After treatment with RNase-free recombinant DNase I, total RNA was subjected to rRNA depletion followed by preparation of cDNA libraries and RNA-seq analysis (transcriptome sequencing) with the Illumina NovaSeq 6000 System sequencing, which were performed by Azenta (Tokyo, Japan). Sequenced reads were generated by base calling with the standard Illumina sequencing pipeline and paired-end RNA-seq data were generated at a read length of 100 bp. The RNA-seq dataset was analyzed with CLC Genomic Workbench software ver. 10 (Qiagen). After mapping the short reads to the reference genome sequences of *S. coelicolor* A3(2) (NCBI accession number: AL645882), differentially expressed genes were identified by the “Differential expression for RNA-seq” tool. The genes with a max group mean > 10, an absolute fold change > 5, and a *p*-value < 0.01 were selected for further analysis.

### 2.4 mRNA transcription analysis via reverse transcription quantitative PCR

To analyze the transcription of the six genes surrounding *ccr1* (*sco1840-45*), *S. coelicolor* A3(2) was pre-cultured in ISP2 medium (Kieser et al., 2000) for 3 days and then mono-cultured or combined-cultured with *T. pulmonis* in YGGS medium for 8 h. The cells were harvested from the culture medium, and RNAprotect Bacteria Reagent (Qiagen) was used to stabilize RNA. After mechanical disruption of the cells using Cell Destroyer (PS-1000, BMS, Tokyo, Japan), total RNA was isolated using RNeasy Mini Kit (Qiagen) following the manufacturer's protocol. After DNA degradation using recombinant DNase I (Takara Bio, Shiga, Japan), an equal quantity of total RNA (500 ng) was used in reverse transcription with PrimeScript RTase (Takara Bio). PCR was performed using GoTaq^®^ Green (Promega, Madison, WI, USA), according to the manufacturer's protocol.

cDNA fragments corresponding to transcripts of the six genes were amplified using specific oligoDNA primers (**Table 3**). The amount of cDNA fragments corresponding to the mRNA for *hrdB* (*sco5820*) was used as a positive control. No amplified products were detected in the control experiments using RNA samples without reverse transcription.

### 2.5 Gene knockout and complementation

Knockout of *ccr1* and *sco1843* from *S. coelicolor* A3(2) and *HEK616_16340* from *S. nigrescens* HEK616 was carried out by CRISPR-base editing systems (Tong et al., 2019). Genome sequencing of *S. nigrescens* HEK616 was previously performed to obtain complete sequences (AP026073 and AP026074) (Asamizu et al., 2022a). Genome annotation of *S. nigrescens* HEK616 was generated by the antiSMASH tool (Blin et al., 2023) to obtain the GenBank file. A 20-nucleotide spacer (Table 1) was designed using CRISPy-web (https://crispy.secondarymetabolites.org/) using the GenBank file to introduce a nonsense mutation in the gene of interest. The plasmid pCRISPR-cBEST, which contains a 20-nucleotide spacer, was introduced into the strains A3(2) and HEK616 via conjugation as previously described (Asamizu et al., 2022a; Lei et al., 2023). Expression of the Cas9–cytidine deaminase fusion protein was induced by adding thiostrepton. Successful base editing was verified using Sanger sequencing.

**Table 1:** Oligonucleotide sequences used in this study.

**Purpose**	**Name**	**Sequence (5^′^to 3^′^)**
RT-qPCR	SCO1840_RTPCRFw	CCGGTGCCCGTGTCCTCATC
	SCO1840_RTPCRRv	CTCACCGGCCAGGACCTTGG
	SCO1841_RTPCRFw	CAGTCTAACGGGGCGGTGCA
	SCO1841_RTPCRRv	GACTGGTCGGGTCCATGGCC
	SCO1842_RTPCRFw	GAATGGCGCGCTCCGGTCTT
	SCO1842_RTPCRRv	GTCGGACAGGGTGGCCTTGC
	SCO1843_RTPCRFw	GCGTGGTTCACCCCCAACCT
	SCO1843_RTPCRRv	GAGTTCCCCCGGGACCTCGA
	SCO1844_RTPCRFw	GACGGACTGGTGGTGGGCAC
	SCO1844_RTPCRRv	GGTCAGCCGGTCGTAGGGGA
	SCO1845_RTPCRFw	ACCATTTCGACCGGTGCGCT
	SCO1845_RTPCRRv	GTGTTGGCGACCTCCACCGA
Gene complementation	sco1842complementFw	TACGAATTCACCCTCCGCCGCTTCATGAC
	sco1842complementRv	CCCAAGCTTGACCAGGACTACGGCTCGCG
Base editing	SCO1842KOspacer	GGTAGGATCGACGGCGACCGCCCAGCTGCCGAGCGGTTTTAGAGCTAGAA
	SCO1842KOspacerc	TTCTAGCTCTAAAACCGCTCGGCAGCTGGGCGGTCGCCGTCGATCCTACC
	SCO1843KOspacer	GGTAGGATCGACGGCGGTGAACCACGCCCGGTCGCGTTTTAGAGCTAGAA
	SCO1843KOspacerc	TTCTAGCTCTAAAACGCGACCGGGCGTGGTTCACCGCCGTCGATCCTACC
	HEK16340KOspacer	GGTAGGATCGACGGCCAGCGCCCAGCCCAGGAGTTGTTTTAGAGCTAGAA
	HEK16340KOspacerc	TTCTAGCTCTAAAACAACTCCTGGGCTGGGCGCTGGCCGTCGATCCTACC
Conformation of base editing by Sanger sequence	Cbestcheck_SCO1842_Fw	GGTGGCCGAGGTCGTCTCCG
	Cbestcheck_SCO1842_Rv	CAGCCACAGCAGCGCGTACG
	Cbestcheck_SCO1843_Fw	TCCTGGACACCGCCGAGCAC
	Cbestcheck_SCO1843_Rv	GAGTCCAGGACGAGCCCCGC
	Cbestcheck_HEK16340_Fw	CGGGCCACGGTCAGCAGTTC
	Cbestcheck_HEK16340_Rv	GGCACTGCCGCAACTGCTCT

Gene complementation was performed using the pTYM1a integration vector (Lei et al., 2023). Briefly, the DNA fragment containing the gene of interest and its upstream region were amplified using primers from the genomic DNA of strain A3(2) (Table 1) and ligated into the corresponding restriction enzyme sites of the plasmid. The generated plasmid was introduced into the strain via *E. coli* ET12567 (pUZ8002)-mediated conjugation. Successfully complemented colonies of mutants were selected using 25 μg/mL apramycin. pTYM1a empty vector was also introduced into the A3(2) parent strain and the knockout strains for control experiments.

### 2.6 Secondary metabolite assay for *S. coelicolor* A3(2)

For RED (undecylprodigiosin) production in liquid culture, parent strain A3(2) and knockout strain Δ*ccr1* were culture for 2 days in a PGA [components (g/L): peptone, 5; glycerol, 10]. The amount of RED was measured according to the previously described protocol (Kieser et al., 2000).

For CDA production, ~1 × 10^5^ spores of each strain were spotted on solid NAHU medium [components (g/L): beef extract, 1; yeast extract, 2; peptone, 5; NaCl, 5; histidine, 0.25; uracil, 0.1; and agar, 15 (pH 7.4)] and grown at 30°C for 48 h (Chong et al., 1998). The plates were then overlaid with 12 mL of soft nutrient agar containing 40 mM Ca(NO_3_)_2_ mixed with 120 μL of an overnight culture of a *Bacillus subtilis* indicator strain and incubated for an additional 48 h. CDA production was compared based on the size of inhibition zones. A CDA-deficient strain, Δ*cdaPS1*, was generated as previously described and used as the negative control (Lei et al., 2023).

For CPK production, ~1 × 10^5^ spores of each strain were spotted on solid 79NG medium [components (g/L): yeast extract, 2; casamino acids, 2; peptone, 10; NaCl, 6; and agar, 20 (pH 7.3)] and grown at 30°C for 24 h (Pawlik et al., 2010). CPK production was compared based on the appearance of yellow pigment in colonies. A CPK-deficient strain, Δ*cpkA*, was generated as previously described and used as the negative control (Lei et al., 2023).

For DES production, ~1 × 10^5^ spores of each strain were spotted on solid medium R2YE and grown at 30°C for 48 h (Craig et al., 2012). The plates were then overlaid with 12 mL of Chrome Azurol S medium (Schwyn and Neilands, 1987) without the addition of nutrients. After overnight incubation at 30°C, DES production was compared based on the size of orange halo zones. A DES-deficient strain, Δ*desA*, was generated as previously described and used as the negative control (Lei et al., 2023).

### 2.7 Secondary metabolite assay for *S. nigrescens* HEK616

Wild-type and mutant *S. nigrescens* HEK616 strains were pre-cultured in ISP2 medium for 3 days and then mono-cultured or combined-cultured with *T. pulmonis* in A3M medium [components (g/L): soluble starch, 20; glucose, 5; glycerol, 20; pharmamedia, 15; yeast extract, 3: Diaion HP-20, 10 (pH 7.2)] for 5 days. Then, the culture broth was extracted with an equal volume of *n*-butanol. After centrifugal evaporation of the solvent, the extract was dissolved in DMSO and analyzed by ultra-performance liquid chromatography-electrospray ionization-quadrupole time-of-flight mass spectrometry (UPLC-ESI-QTOF/MS) (Agilent Technologies, Waldbronn, Germany). Mass spectrometry settings are described in our previous papers (Asamizu et al., 2022a,b). Chromatographic separation was performed with a reverse phase column using a BEH C18 2.1 × 30 mm column (Waters Corp., Milford, MA, USA) with a flow rate of 0.5 mL/min and the following gradient of solvent A (0.1% formic acid in H_2_O) to solvent B (acetonitrile): *t* = 0–0.5 min, maintain B at 5%; *t* = 0.5–8.5 min, increase B to 95%; *t* = 8.5–11.5 min, maintain B at 95%; *t* = 11.5–12 min, decrease B to 5%.

### 2.8 Bioinformatics analysis

Evolutionary analyses were conducted in MEGA11 (Tamura et al., 2021). The evolutionary history was inferred using the maximum likelihood method and Jones et al. w/freq. model. The bootstrap consensus tree inferred from 1,000 replicates was considered to represent the evolutionary history of the taxa analyzed. Branches that were reproduced in fewer than 50% bootstrap replicates were collapsed. The percentage of replicate trees in which the associated taxa clustered together are shown next to the branches. Initial tree(s) for the heuristic search were automatically obtained by applying neighbor-joining and BioNJ algorithms to a matrix of pairwise distances estimated using the JTT model; then, the topology was selected by the superior log likelihood value. A discrete Gamma distribution was used to model evolutionary rate differences among sites [5 categories (+G, parameter = 0.6296)]. This analysis involved 173 amino acid sequences. There were a total of 849 positions in the final dataset.

The aligned gene map was drawn by Clinker (https://github.com/gamcil/clinker) (Gilchrist and Chooi, 2021). Geneious Prime (Dotmatics, Boston, MA, USA) was used to analyze the Sanger sequence data. Multiple alignment was performed by ClustalW (https://www.genome.jp/tools-bin/clustalw) and drawn by jalview (https://www.jalview.org/) (Clamp et al., 2004). Predicted protein 3D structure ribbon models were drawn by ChimeraX (https://www.cgl.ucsf.edu/chimerax/) (Meng et al., 2023).

## 3 Results

### 3.1 Identification of point mutations in Mt-206005 by genome re-sequencing

**Figure 1:**
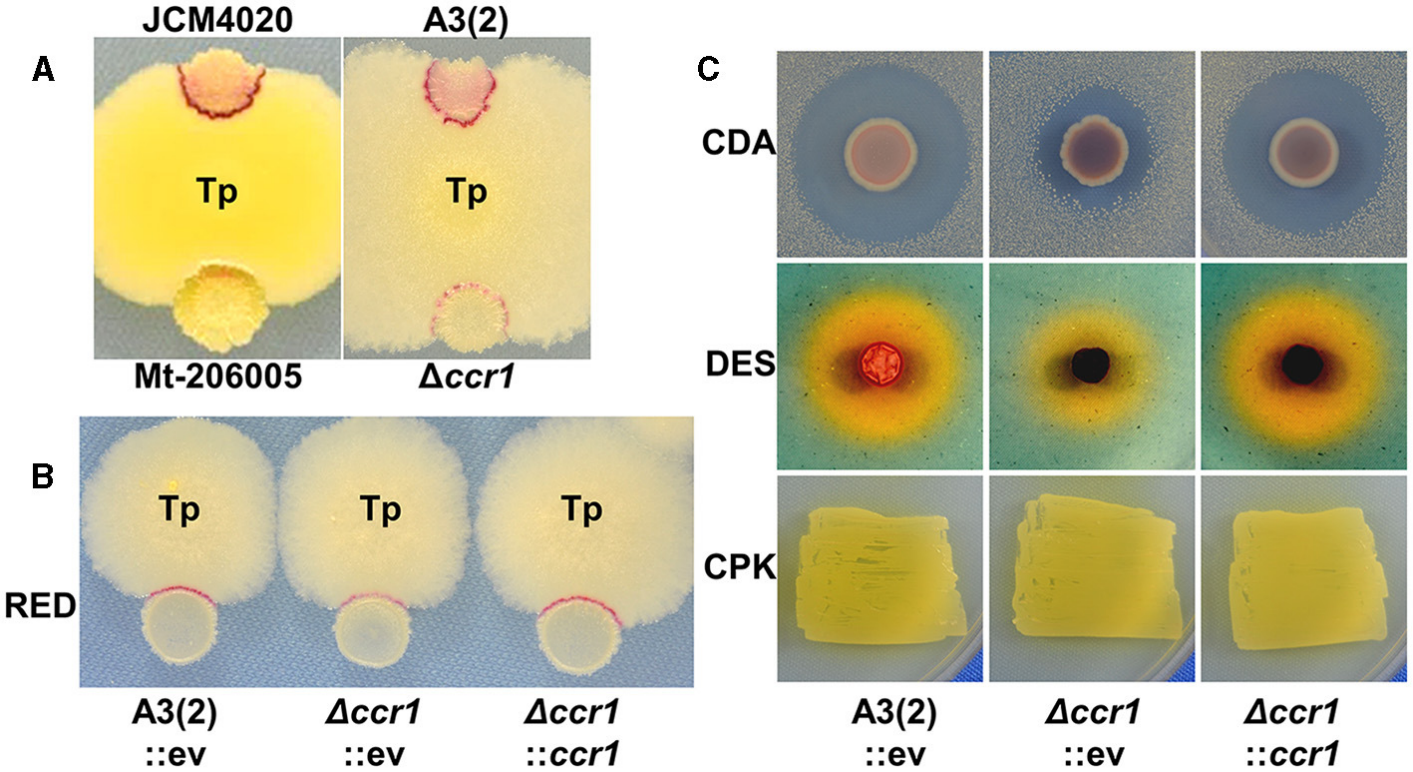
Impact of *ccr1* (*sco1842*) inactivation on secondary metabolite production. **(A, B)** Competitive cultures (day 4) were performed in YGGS medium. Tp, *T. pulmonis* TP-B0596; A3(2), *S. coelicolor* A3(2); RED, undecylprodigiosin; CDA, calcium-dependent antibiotic; DES, desferrioxamine; CPK, cryptic polyketide; ev, empty vector. **(C)** CDA production was evaluated by the size of the *B. subtilis* growth inhibition halo on NAHU medium. DES production was evaluated by the halo size based on colorimetric change of CAS reagent on R2YE medium. CPK production was evaluated by a yellowish color of the colony on 79NG medium.

Previously, we generated a mutant library using wild-type strain of *S. coelicolor* JCM4020 by carbon-ion (^12^C^5+^) beam irradiation, and subsequently screened for mutants deficient in RED production under interaction with *T. pulmonis* TP-B0596 (Yanagisawa et al., 2022). Among the selected mutants, Mt-206005 exhibited a deficient RED production phenotype (Figure 1A). To identify the genes responsible for deficient RED production, we conducted a detailed analysis of point mutations induced in the genome of Mt-206005, which displayed reduced RED production upon interaction with *T. pulmonis*. Among four identified point mutations, the *JCM4020_19880* gene harbored a mutation (1122delG) that resulted in a frameshift (Pro341FS), which may disrupt the function of the protein (Table 2). The *JCM4020_19880* product displayed 100% identity with the *sco1842* (*ccr1*) gene product in the model actinomycete *S. coelicolor* A3(2). Therefore, for further analyses, we used *S. coelicolor* A3(2) M145, which has identical genes, and analyzed the involvement of *ccr1* in SM production.

**Table 2:** Point mutations in Mt-206005.

**Genomic region**		**Nucleotid mutation**	**Amino acid mutation**	**Putative function of deduced protein**	**SCO homolog**
2056643	JCM4020_19480	416C>T	Ser139Phe	L-fuco-beta-pyranose dehydrogenase (EC 1.1.1.122)	*sco1803*
2095521	JCM4020_19880	1122delG	Pro341FS	Hypothetical protein	*sco1842* (*ccr1*)
2434326	JCM4020_22910	1165G>T	Gly389Cys	Two component sensor kinase	*sco2142*
3652515	JCM4020_33590	892C>G	Asp298His	Molybdopterin biosynthesis protein MoeA	*sco3181*

### 3.2 Δ*ccr1* showed reduced SM production

To determine if *ccr1* inactivation induces reduced RED production in *S. coelicolor* A3(2) by interaction with *T. pulmonis*, we generated a targeted gene knockout mutant, Δ*ccr1* (Table 3; Supplementary Figure S1). *ccr1* encodes a protein with 447 amino acids that was annotated as a hypothetical protein. Notably, *ccr1* was neither located inside nor in close proximity to known gene clusters for SMs in *S. coelicolor* A3(2) (Nett et al., 2009). Δ*ccr1* did not show noticeable phenotypic changes, including sporulation in MS medium (Supplementary Figure S2). Additionally, when interacting with *T. pulmonis*, the Δ*ccr1* mutant exhibited reduced RED production comparable to that of Mt-206005 (Figures 1A, B). Notably, RED production was also slightly diminished in mono-culture growing on PGA medium (Supplementary Figure S2). The amount of RED was measured in both mono- and combined-culture in liquid and was consistent with what was observed in the agar culture (Supplementary Figure S3). This indicates that *ccr1* is not the sole factor in mediating the signal from *T. pulmonis* but does affect the basic production of RED. We further tested *ccr1* overexpression under the ermE^*^ constitutive promoter in *S. coelicolor* A3(2) to assess whether it could enhance RED production. However, it did not have an apparent effect on RED production (data not shown).

**Table 3:** Genome editing to introduce a TAA stop codon.

**Gene**	**Guide RNA sequence (in antisense DNA sequence)**	**PAM**	**Amino acid mutation caused by C to T mutations**
*sco1842* (*ccr1)*	5′-GACCGCCCAGCTGCCGAGCG	GGG-3′	W242^*^
*sco1843*	5′-GGTGAACCACGCCCGGTCGC	CGG-3′	W115^*^
*HEK_16340*	5′-CAGCGCCCAGCCCAGGAGTT	CGG-3′	W223^*^

^*^indicates the stop codon.

We also assessed the productivities of other SMs, including calcium-dependent antibiotics (CDAs) (Hojati et al., 2002), desferrioxamines (DESs) (Barona-Gomez et al., 2004), and cryptic polyketides (CPKs) (Gomez-Escribano et al., 2012), in Δ*ccr1* through dedicated agar plate assays for mono-culture (Figure 1C). Although the apparent CPK productivity remained unchanged in Δ*ccr1*, we observed reduced productivity for both CDAs and DESs; this indicated that the function of *ccr1* gene products extends beyond RED production and affects a broader spectrum of SM production. Furthermore, the phenotypes of Δ*ccr1* for SM production were effectively restored by gene complementation *in trans* to the *attB* site of phiC31 integrase (Figures 1B, C).

### 3.3. Ccr1 is a highly conserved hypothetical protein in *Streptomyces* species

**Figure 2:**
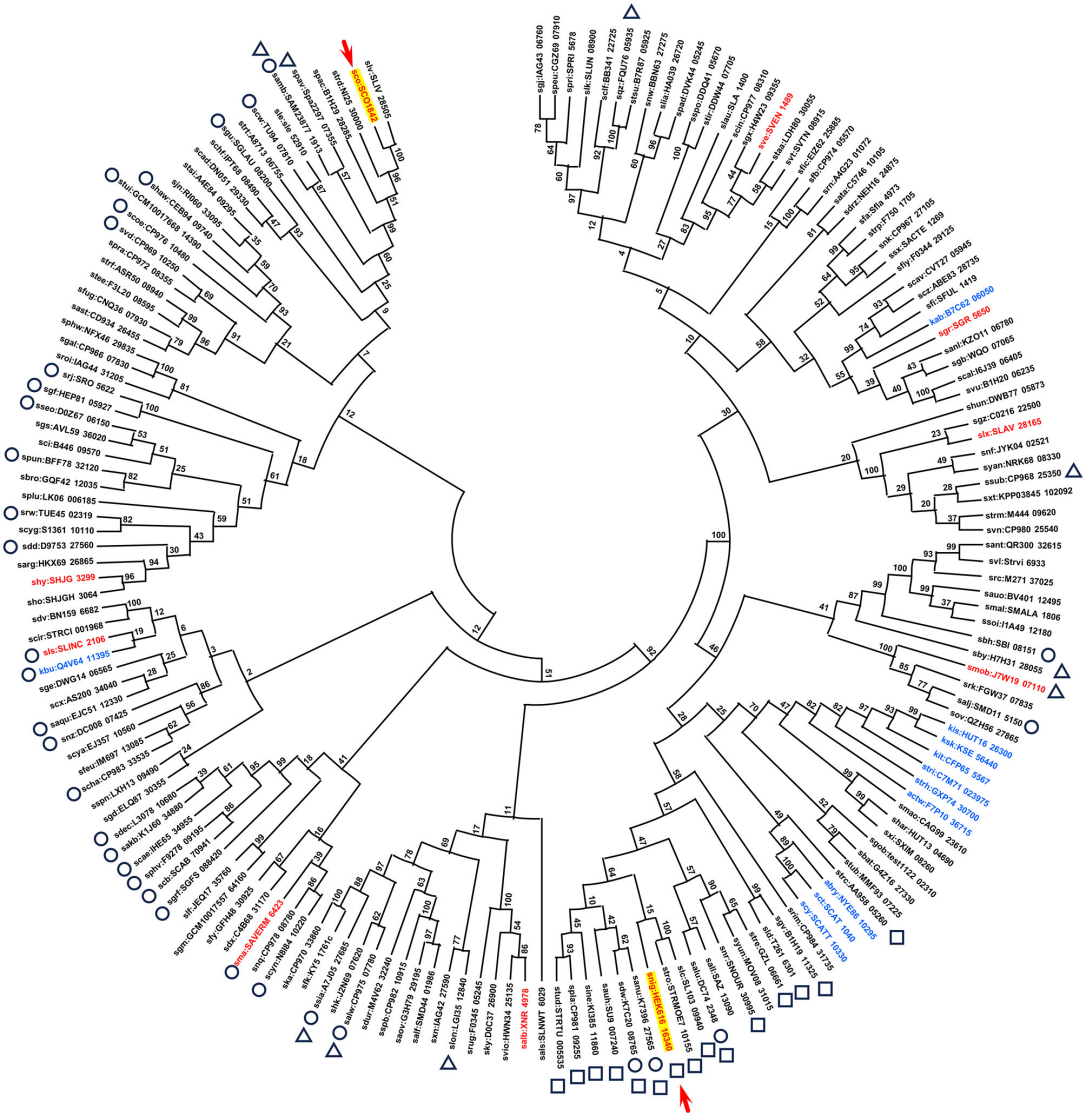
Phylogenetic tree of Ccr1 homologs. The top 173 scoring proteins found by BLAST search of the KEGG database were used to construct the phylogenetic tree by maximum likelihood analysis. The position of SCO1842 (Ccr1) and HEK_16340 (Ccr1^HEK616^) is indicated by a red arrow. Circle: 33 proteins that contain a HTH motif; square: 15 proteins that contain a HEAT motif; triangle: nine proteins that contain a HCTD (Helicase C-terminal Domain) motif. Details are listed in Supplementary Table S2. Non-*Streptomyces* strains are indicated in blue. The flanking regions of the corresponding genes, indicated in red, were selected and presented in the Figure 5.

**Figure 3:**
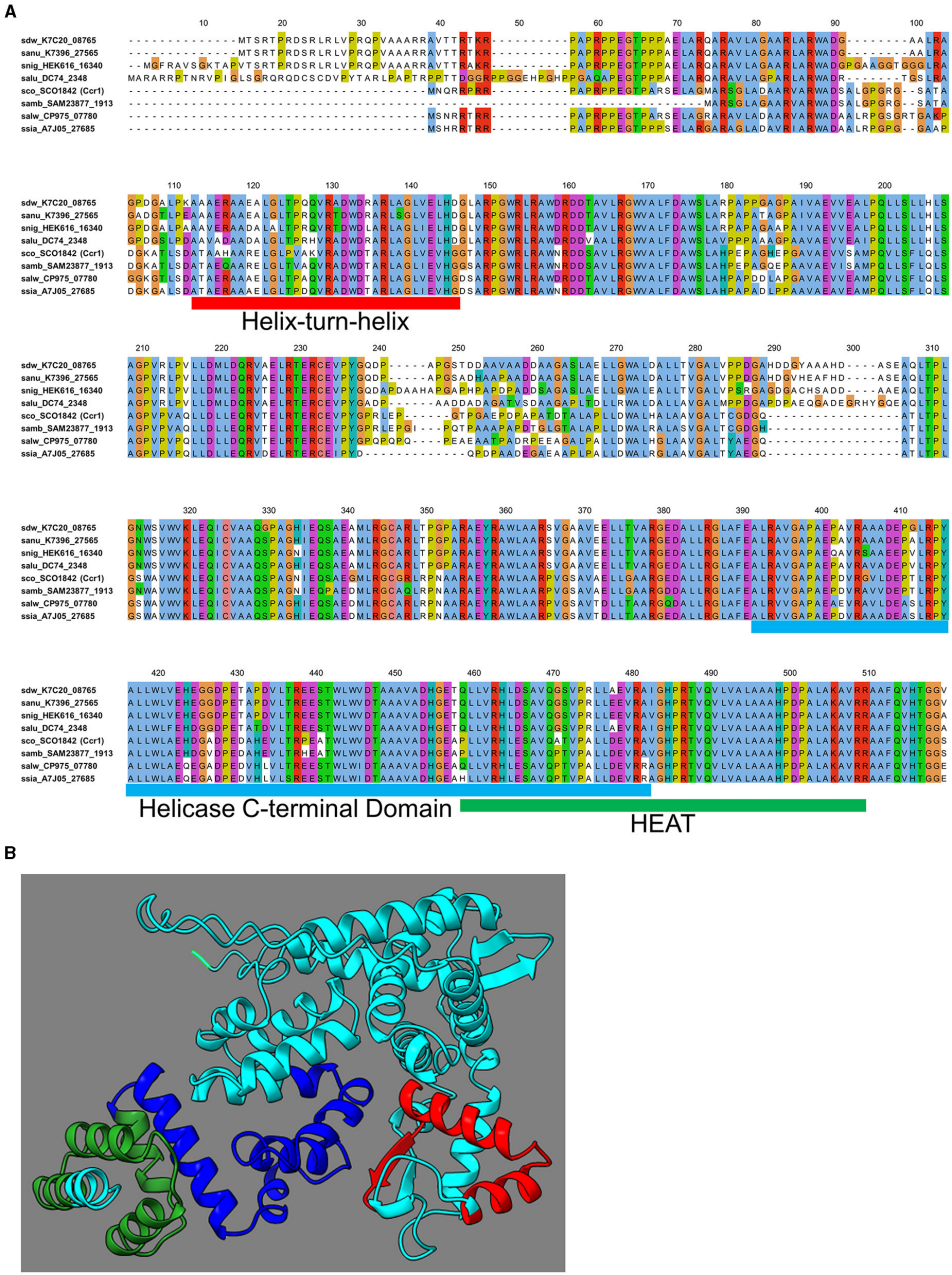
Multiple alignments of Ccr1 homologs and putative 3D structure of Ccr1. **(A)** Ccr1 homologs containing HTH/HCTD (A7J05_27685, CP975_07780, SAM23877_1913) motifs or HTH/HEAT (DC74_2348, K7396_27565, K7C20_08765) motifs were aligned by ClustalW with Ccr1 (SCO1842) and Ccr1^HE616^ (HEK616_16340). **(B)** Putative 3D structure of Ccr1 in ribbon model predicted by Alphafold2. The HTH motif is indicated in red; the HCTD motif is indicated in blue; the HEAT motif is indicated in green.

The Ccr1 amino acid sequence from *S. coelicolor* A3(2) was used for BLAST search of the Kyoto Encyclopedia of Genes and Genomes (KEGG) database (Kanehisa et al., 2017). A total of 169 putative proteins exhibited scores exceeding 300 (e-value: > 5 × 10^−101^) (Supplementary Table S2). The KEGG database includes genomes of 165 *Streptomyces* species (to date); among the 169 homologs identified, 159 (/165, 96.4%) originated from different *Streptomyces* species (Figure 2; Supplementary Table S2). Although the function of Ccr1 remains entirely unknown, the high conservation of its homolog in *Streptomyces* species underscores the gene's fundamental importance. Although the amino acid sequence of Ccr1 did not display any conserved motifs or domains, we found that some of its homologous proteins contained slight signatures for a helix–turn–helix (HTH) motif in the N-terminal region (33/169), and a helicase conserved C-terminal domain (HCTD) (9/169) or a HEAT (15/169) motif at the C-terminal region (Supplementary Figure S4, Supplementary Table S2). Generally, the HTH is involved in DNA binding into the major groove, where the recognition helix makes most DNA contacts (Aravind et al., 2005). DNA helicases catalyze the unwinding of double-stranded DNA to yield the single-stranded DNA intermediates required in DNA replication, recombination, and repair (Lohman and Bjornson, 1996). HEAT repeats are structural architecture, and HEAT-containing proteins are involved in a multitude of cellular processes, including intracellular transport, signaling, and protein synthesis (Friedrich et al., 2022). Multiple alignment of Ccr1 and the seven other selected homologs possessing putative motifs showed that amino acid sequences of HTH, HCTD, or HEAT motifs were relatively conserved among the homologs (Figure 3A). This result indicated a conserved function of this protein.

Additionally, 3D structure of Ccr1 was predicted by AlphaFold2 (ColabFold) (Figure 3B; Supplementary Figure S5) (Jumper et al., 2021; Mirdita et al., 2022). Overall, the predicted motif structures of HTH, HCTD, or HEAT were conserved among the homologous proteins in putative 3D structures (Supplementary Figure S4). These findings suggested that Ccr1 and other homologs, including those that do not show the clear motifs, also contain these putative motifs.

### 3.4 RNA sequence analysis of the Δ*ccr1* mutant revealed genome-wide variation of gene transcription

RNA sequence analysis was conducted on strains A3(2) and Δ*ccr1* to gain insights into the comprehensive impact of *ccr1*. Transcriptome analysis often reveals significant redundancy in transcriptional variations, especially when comparing phenotypically distinct strains. Therefore, we performed RNA-seq analysis on the A3(2) parent strain and the Δ*ccr1* mutant, both of which exhibited similar phenotypes on agar plates. Using 79NG medium, we observed comparable phenotypes between A3(2) and Δ*ccr1* (Supplementary Figure S2).

The RNA-seq results indicated that 253 genes (with 7846 coding sequences) exhibited a more than 5-fold differential transcription in the Δ*ccr1* mutant compared with A3(2) (Supplementary Table S3), with 148 genes showing decreased and 105 genes showing increased transcription. The substantial variation in gene transcription was intriguing, particularly considering the unchanged phenotype of the growing colony. However, this was anticipated based on the predicted motifs in Ccr1 and suggested by its global effects. Among the genes showing variation, several clustered regions displayed consistent changes in the Δ*ccr1* mutant (Supplementary Table S3). Genes in the actinorhodin (ACT) biosynthesis cluster (*sco5071-92*) (Nett et al., 2009) showed down-regulation in the Δ*ccr1* strain. Furthermore, although the relevance is not clear, genes *sco0162-81*, which belong to the regulon of the DevR involved in nitric oxide (NO) signaling and NO homeostasis (Urem et al., 2016), were down-regulated in the Δ*ccr1* strain. Of the SM BGCs, we only observed up-regulation in coelichelin (*sco0498-0499*) and 5-hydroxyectoine (*sco1865*) (Nett et al., 2009).

### 3.5 Functional analysis of a *ccr1* homolog (*HEK616_16340*) in *S. nigrescens* HEK616

To investigate whether the Ccr1 homologs are functionally conserved, we tested the impact of gene knockout of the *ccr1* homolog gene *HEK616_16340* in another strain, *S. nigrescens* HEK616 (Table 2; Supplementary Figure S1). The HEK616 strain was isolated from soil samples of Hegura Island, Ishikawa in Japan as part of the HEK strain series (Kato et al., 2022). The gene product of *ccr*1^HEK616^ (*HEK616_16340*) exhibited 63.7% identity to Ccr1 (SCO1842) [identity: 323/507, similarity: 350/507 (69.0%), gaps: 63/507 (12.4%)] (Figure 3A). Additionally, the 3D structure of Ccr1^HEK616^ predicted by AlphaFold2 was conserved relative to those of Ccr1 and other homologs (Supplementary Figure S5). Strain HEK616 produced streptoaminals and 5aTHQs (Supplementary Figure S6) in combined-culture with *T. pulmonis* (Sugiyama et al., 2015, 2016), and both compounds were predicted to originate from the common *stm* BGC, which consisted of nine genes (*HEK616_65060-64980*) (Ozaki et al., 2019). The *stm* genes were also not flanked by *ccr*1^HEK616^.

**Figure 4:**
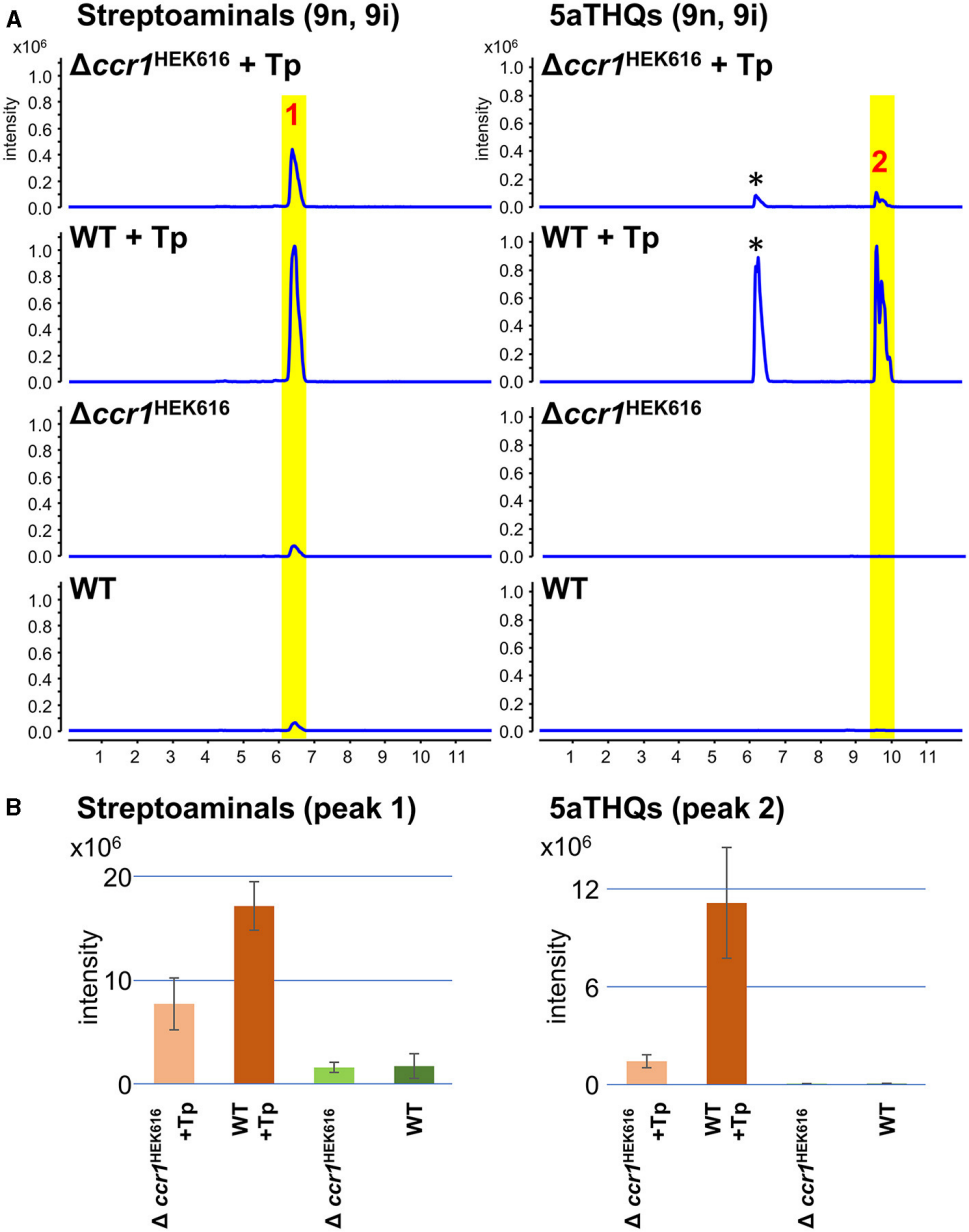
Impact of *ccr1* homolog on production of streptoaminals and 5aTHQs in strain HEK616. **(A)** Mono- or combined-cultures of HEK616 wild-type (WT) and Δ*ccr*1^HEK616^ (*HEK_16340*) were performed on A3M medium for 5 days. Peak 1 contained a mixture of streptoaminals-9i and 9n and peak 2 contained a mixture of 5aTHQs-9i and 9n; chemical structures are shown in Supplementary Figure S6. Peaks indicated by asterisks are uncharacterized metabolites with similar *m/z* values (33). Tp, *T. pulmonis* TP-B0596. **(B)** Quantification of streptoaminals and 5aTHQs based on the peak area of the extracted ion chromatogram (*n* = 3).

Strains HEK616 (wild-type) and Δ*ccr*1^HEK616^ were combined-cultured with *T. pulmonis* in A3M production medium. In the combined-culture, UPLC-ESI-QTOF/MS analysis of the culture extract revealed specific and enhanced production of 5aTHQs (-9i, 9n) and streptoaminals (-9i, 9n), respectively, in the wild-type strain (Figures 4A, B), which was consistent with previous findings (Sugiyama et al., 2015, 2016). In contrast, the Δ*ccr*1^HEK616^ strain exhibited significantly reduced production of 5aTHQs and streptoaminals in combined-culture (0.13- and 0.45-fold, respectively; Figures 4A, B). In mono-culture, a relatively small amount of streptoaminals was detected in the mono-culture extract, and this portion of production in mono-culture was also slightly diminished in the Δ*ccr*1^HEK616^ strain (Figures 4A, B). This finding was consistent with those of our previous reports (Sugiyama et al., 2015, 2016; Ozaki et al., 2019). These results demonstrate that *ccr*1^HEK616^ is also involved in SM production, which indicates that the function of the Ccr1 homolog in SM production regulation is conserved across *Streptomyces* species.

### 3.6 *ccr1* and *sco1843* were up-regulated in combined-culture

**Figure 5:**
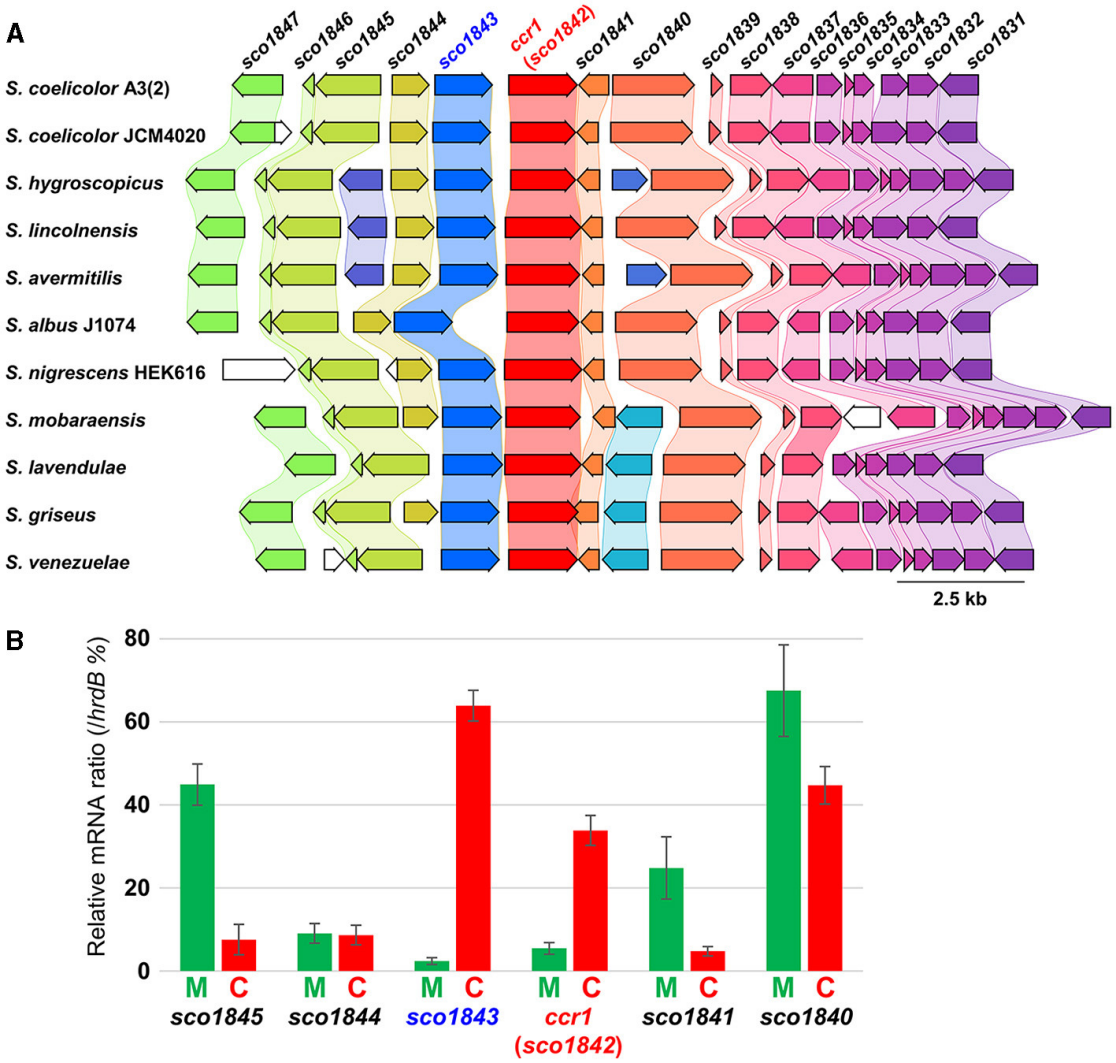
Gene map of the conserved *ccr1* flanking region and RT-qPCR analysis. **(A)** *S. coelicolor* A3(2) is a model strain used in this study; *S. hygroscopicus* subsp. *jinggangensis* 5008 is a validamycin producer; *S. lincolnensis* NRRL 2936 is a lincomycin producer; *S. avermitilis* MA-4680 is an avermectin producer; *S. albidoflavus* (*albus*) J1074 is a general host for heterologous SM production; *S. nigrescens* HEK616 is a streptoaminal and 5sTHQ producer used in this study; *S. mobaraensis* NBRC 13819; *S. lavendulae* subsp. *lavendulae* CCM 3239 is a bleomycin producer. *S. griseus* subsp. *griseus* NBRC 13350 is a streptomycin producer and a model strain used for biological study; *S. venezuelae* ATCC 10712 is a chloramphenicol producer and a model strain used for biological study. **(B)** The green bar indicates the mono-culture (M), and red bar indicates the combined-culture (C). The values were normalized by the mRNA level of the housekeeping gene *hrdB*.

Although the Δ*ccr1* mutant itself exhibited reduced RED production, the effect of the *ccr1* homologs in the combined-culture of both strains A3(2) and HEK6161 was significant. Therefore, we further analyzed gene transcription using RT-qPCR in both mono-culture and combined-culture using strain A3(2). We conducted transcription analyses of *ccr1* and the flanking region genes. The flanking region contained genes encoding an ABC transporter ATP-binding protein (*sco1840*), a hypothetical protein with glyoxalase motif (*sco1841*) in the downstream region, and a hypothetical protein with hydrolase motif (*sco1843*), a L-fuculose-phosphate aldolase (*sco1844*), and an inorganic phosphate transporter (*sco1845*) in the upstream region (Figure 5A). Remarkably, *ccr1* transcription significantly increased by more than 6.2-fold in the combined-culture (Figure 5B; Supplementary Table S4). This observation is remarkable and suggests the involvement of the gene (product) in global regulation of SMs that results from bacterial interaction. Moreover, even though the transcription of other genes (*sco1840-41, sco1844-45*) in the flanking region were not up-regulated, the *sco1843* gene exhibited 26.8-fold up-regulation in the combined-culture (Figure 5B; Supplementary Table S4). In the RNA-seq data comparing the parent strain and Δ*ccr1*, only the transcription of the *sco1843* gene showed a significant fold change (0.36-fold, *p*-value 0.01). The other examined genes (*sco1840, sco1841, sco1844*, and *sco1845*) did not show significant validation. Additionally, it is known that SCO1845 encodes a putative low-affinity Pi transporter, PitH2, and the expression of *pitH2* is dependent on the response regulator (RR) of the two-component system (TCS) PhoP (Santos-Beneit et al., 2008). PhoP binds to specific sequences consisting of direct repeats of 11 nt in the promoter of *pitH2*. Considering these facts, a functional association of *sco1843* and *ccr1* was predicted; therefore, we further analyzed the impacts of *sco1843* on SM production.

Conservation of *sco1843* homologs were searched using the KEGG database. Most of the strains that possess *ccr1* contained adjacent *sco1843* homologs (167/169) (Figure 5A; Supplementary Table S5). Our results indicate functional relationships between *ccr1* and *sco1843* (e.g., up-regulation in combined-culture, gene adjacency, and gene conservation). The transcript pattern in the RNA-seq showed two distinct peaks and there is a 402 bp intergenic region between the two genes, we consider that *ccr1* (*sco1842*) and *sco1843* are not co-transcribed as a single cistron. Nevertheless, we generated a knockout strain of *sco1843* to examine the effect on phenotype, including RED production (Table 2; Supplementary Figure S1). We examined RED production in both mono-culture and combined-culture. However, no apparent change in RED production was observed in either by *sco1843* inactivation (Supplementary Figure S7). Aside from RED production, the apparent phenotype also did not show differences between A3(2) and the Δ*sco1843* mutant in six different agar culture conditions, including sporulation on mannitol soya flour (MS) medium (Supplementary Figure S2). Despite the implications that there is a functional relationship between the two genes, the results revealed that *sco1843* did not have an apparent cooperative role with *ccr1* for RED synthesis.

## 4 Discussion

In this study, building upon insights gained from forward genetic investigations, we identified *ccr1* as the causative factor for production of several SMs, including RED, CDA, DES, and ACT in *S. coelicolor* A3(2). Importantly, the homologous gene in *S. nigrescens* HEK616 was also demonstrated to be crucial for production of streptoaminals/5aTHQs in combined-culture. Additionally, the importance of the *ccr1* gene in bacterial interaction for the induction of secondary metabolism has also been suggested by the transcriptional activation of the gene in combined-culture with *T. pulmonis*.

Although regulation of streptoaminals/5aTHQs in *S. nigrescens* HEK616 remains unknown, production of RED, CDA, and DES in *S. coelicolor* A3(2) has been relatively well-characterized. There are several other regulatory factors that are known to be involved in RED synthesis of *S. coelicolor* A3(2), including well-characterized *Streptomyces* antibiotic regulatory protein-type cluster-situated regulators (Williamson et al., 2006).

Compared with characterized regulatory systems, Ccr1 has a highly unique primary structure that could not be classified in a known protein family. BLAST search revealed that Ccr1 is highly conserved among *Streptomyces* species, which are soil-dwelling filamentous Gram-positive bacteria. Interestingly, homolog conservation was notably limited to *Streptomyces* species and phylogenetically close *Kitasatospora* species, but was not present in other bacterial species with whole genome sequences in the KEGG database. The specific conservation of this homolog in filamentous growing/spore-forming bacteria suggests the involvement of *ccr1* in hyphal extension growth and/or morphological differentiation. Although more detailed analysis may be required, *ccr1* inactivation did not show a significant impact on apparent morphological differentiation in the tested strains A3(2) and HEK616, as judged by growing colonies on several agar plate conditions (Supplementary Figure S2), which indicated that the observed effect was somewhat significant to SM production.

In addition to the effects on SM production, our RNA-seq data revealed significant variations (more than 5- or < 0.2-fold) in the expression of more than 100 genes, both up- and down-regulated, in mono-culture. The impact of Ccr1 appeared to be pleiotropic, affecting not only specific regions or genes, but exerting a more widespread influence on the genome, as indicated by RNA-seq analysis. Ccr1 exhibited a slight but discernible signature for a HTH motif in its N-terminal region, which indicated a potential association with DNA and a role in gene expression regulation. A substantial number of transcriptome analyses have been conducted on *S. coelicolor* A3(2) under various conditions (Jeong et al., 2016). It is worth noting that, to our knowledge, *ccr1* has not been mentioned in genome-wide omics studies. This indicates that *ccr1* has a unique function and it may be involved in specific bacterial interaction, such as with *T. pulmonis*. You may see the explanation of the genes in the Supplementary material, which showed variation and have been reported in the literature (Supplementary Table S3).

Nucleoid-associated proteins (NAPs) are generally characterized as small, abundant transcriptional regulators with low sequence specificity that participate in diverse DNA-related processes such as gene expression, DNA protection, recombination/repair, and nucleoid structuring. In *Streptomyces* species, a well-explored family of NAPs includes BldC. BldC (SCO4091) is a compact MerR-like protein (68 amino acids) featuring an HTH motif (Dorman et al., 2020). Mutations in BldC lead to premature development. BldC is recognized for its role in delaying entry into development; it fosters prolonged vegetative growth by binding to numerous promoter regions. Schumacher et al. (2018) discovered that BldC forms identical head–tail dimers, with multiple subunits cooperatively binding to DNA, which causes distortion and shortening of its structure.

Aside from BldC, Bradshaw et al. conducted a proteomic study to identify NAPs in *S. coelicolor* A3(2) grown in a rich liquid culture medium. Their investigation revealed 24 proteins, including major NAPs such as HupA (SCO2950) (Salerno et al., 2009; Strzalka et al., 2022), HupS (SCO5556) (Szafran et al., 2021), sIHF (SCO1480) (Yang et al., 2012; Swiercz et al., 2013), and Lsr2 (SCO3375) (Gehrke et al., 2019; Zhang et al., 2021). Du et al. proposed Gbn (SCO1839) as a new NAP family, and their omics analysis revealed its genome-wide DNA binding capacity (Du et al., 2022). Despite the proximity of *sco1839* to *ccr1*, knockout experiments showed no mutual impact on transcription, which indicated independent regulation and functions of Gbn and Ccr1. The Ccr1 C-terminal HTH motif also did not exhibit sequence similarity to other characterized NAPs, which is typical for NAPs.

Ccr1 also features a HCTD motif in its C-terminal region. Although the exact function of HCTD is unclear, helicase-like proteins that contain HCTD motif mostly contain DEAD/DEAH box motif, which is responsible for ATP-dependent helicase activity that is absent from Ccr1; this indicates that Ccr1 does not contain helicase activity. Compared with NAPs, limited information is available regarding the function of helicase-like proteins in *Streptomyces* species. The helicase-like protein HelR, which contains UvrD-like ATP-binding domain and is widely distributed in actinobacteria, is induced by rifamycins in *S. venezuelae* (Surette et al., 2022). HelR imparts broad-spectrum rifamycin resistance by forming a complex with RNA polymerase, subsequently ejecting rifamycins. The involvement of helicase-like proteins in SM production has not been described elsewhere. As Ccr1 contains HCTD-like or HEAT-like motifs, the function of these motifs will be investigated in a future project.

The predicted genome-wide effect of the Ccr1 protein suggested by the RNA-seq analysis is noteworthy. However, the observed phenotypic change was confined to SM production rather than influencing cell growth, which may require more detailed analysis. *ccr1* up-regulation in combined-culture indicates its potential association with bacterial interactions. Exploring *ccr1* and its homologs in other *Streptomyces* species could offer valuable insights into their ecological roles during these interactions, which remain largely unknown. This discovery indicates the presence of a novel regulatory protein family and points to a new mechanism that plays a role in SM production in *Streptomyces* species involving bacterial interaction. Understanding this mechanism is an ongoing challenge and offers potential avenues for further exploration in the field of SM regulation.

## Data availability statement

The datasets presented in this study can be found in online repositories. The names of the repository/repositories and accession number(s) can be found in the article/Supplementary material.

## Author contributions

YL: Data curation, Formal analysis, Investigation, Validation, Visualization, Writing – original draft. HO: Conceptualization, Funding acquisition, Methodology, Project administration, Resources, Supervision, Validation, Writing – review & editing. SA: Conceptualization, Data curation, Formal analysis, Funding acquisition, Investigation, Methodology, Project administration, Resources, Supervision, Validation, Visualization, Writing – original draft, Writing – review & editing.
