# Supplementary material for: Transcriptionally induced nucleoid-associated protein-like ccr1 in combined-culture serves as a global effector of Streptomyces secondary metabolism
Source: Front Microbiol. 2024 Jul 12;15:1422977. doi: 10.3389/fmicb.2024.1422977 (PMC11272600; doi:10.3389/fmicb.2024.1422977)
Supplement: Supplementary file 1 [file Data_Sheet_1.zip › Data Sheet 1.PDF]

Supplementary Material

Contents

Table S1. Natural products isolated from combined-culture with *Tsukamurella pulmonis* TP-B0596 ..... **2**
Figure S1. Sanger sequence to confirm the genome editing **3**
Figure S2. Phenotype of Δ*sco1842* and Δ*sco1843* mutants grown on different agar medium **4**
Figure S3. Quantification of RED (undecylprodigiosin) **5**
Table S2. SCO1842 homologs with putative conserved motifs **5-10**
Figure S4. Predicted motifs of SCO1842 homologs found in the KEGG database **11**
Figure S5. Predicted 3D structure of SCO1842 homologs by AlfaFold2 (ColabFold) **12**
Table S3. List of differentially expressed genes in the RNA-seq analysis of Δ*sco1842*. (You can find in an additional supplemental data) **13-14**
Figure S6. Chemical structures of streptoaminals (9i, 9n), and 5aTHQ (9i, 9n) **15**
Figure S7. Impact of *sco1843* on RED production. **15**
Table S4. RT-qPCR analysis of *sco1842* and genes in flanking region **16**
Table S5. Conservation of SCO1843 homologs **16-18**
Table S6. Streptomyces strains that do not possess SCO1842 or SCO1843 homologs **19**
References **19-21**

Table S1. Natural products isolated from combined-culture with *Tsukamurella pulmonis* TP-B0596.

strain	induced natural products	original producing strain	references
Actinosynnema mirum NBRC 14064	mirilactams C-E		(Hoshino et al., **2018b**)
Amycolatopsis sp. 26-4	amycolapeptins A and B		(Pan et al., **2021**)
	amoxetamide A		(Pan et al., **2023**)
Catenuloplanes sp. RD067331	catenulobactins A and B		(Hoshino et al., **2018a**)
Micromonospora wenchangensis HEK797	dracolactams A and B		(Hoshino et al., **2017**)
Saccharothrix sp. A1506	saccharothriolide C-2		(Jiang et al., **2019**)
Streptomyces albogriseolus HEK740	streptogramin B and L-156,587		(Oku et al., **2021**)
Streptomyces cinnamoneus NBRC 13823	arcyriaflavin E		(Hoshino et al., **2015c**)
Streptomyces davawensis JCM 4913	desferrioxamine derivatives		(Hagihara et al., **2018**)
Streptomyces endus S-522	alchivemycin A and B		(Igarashi et al., **2010**)
Streptomyces hygroscopicus HOK021	harundomycin A		(Asamizu et al., **2022**)
Streptomyces nigrescens HEK616	streptoaminals		(Sugiyama et al., **2016**)
	5-alkyl-1,2,3,4-tetrahydroquinolines		(Sugiyama et al., **2015**)
Streptomyces sp. CJ-5	chojalactones A-C		(Hoshino et al., **2015b**)
Streptomyces sp. KUSC_F05	longicatenamides A-D		(Jiang et al., **2021**)
Streptomyces sp. NZ-6	niizalactams A-C		(Hoshino et al., **2015a**)
Umezawaea sp. RD066910	umezawamides		(Hoshino et al., **2018c**)
heterologous expression in *S. lividans* TK23	goadsporin A-C	from *Streptomyces* sp. TP-A0584	(Onaka et al., **2015;** Ozaki et al., **2016**)
heterologous expression in *S. lividans* TK23	staurosporin	from *Streptomyces* sp. TP-A0274	(Onaka et al., **2015**)
heterologous expression in *S. lividans* TK23	rebeccamycin	from *Lechevalieria aerocolonigenes* ATCC 39243	(Onaka et al., **2015**)
heterologous expression in *S. lividans* TK23	streptoaminals	from *Streptomyces nigrescens* HEK616	(Ozaki et al., **2019**)
heterologous expression in *S. lividans* TK23	5-alkyl-1,2,3,4-tetrahydroquinolines	from *Streptomyces nigrescens* HEK616	(Ozaki et al., **2019**)

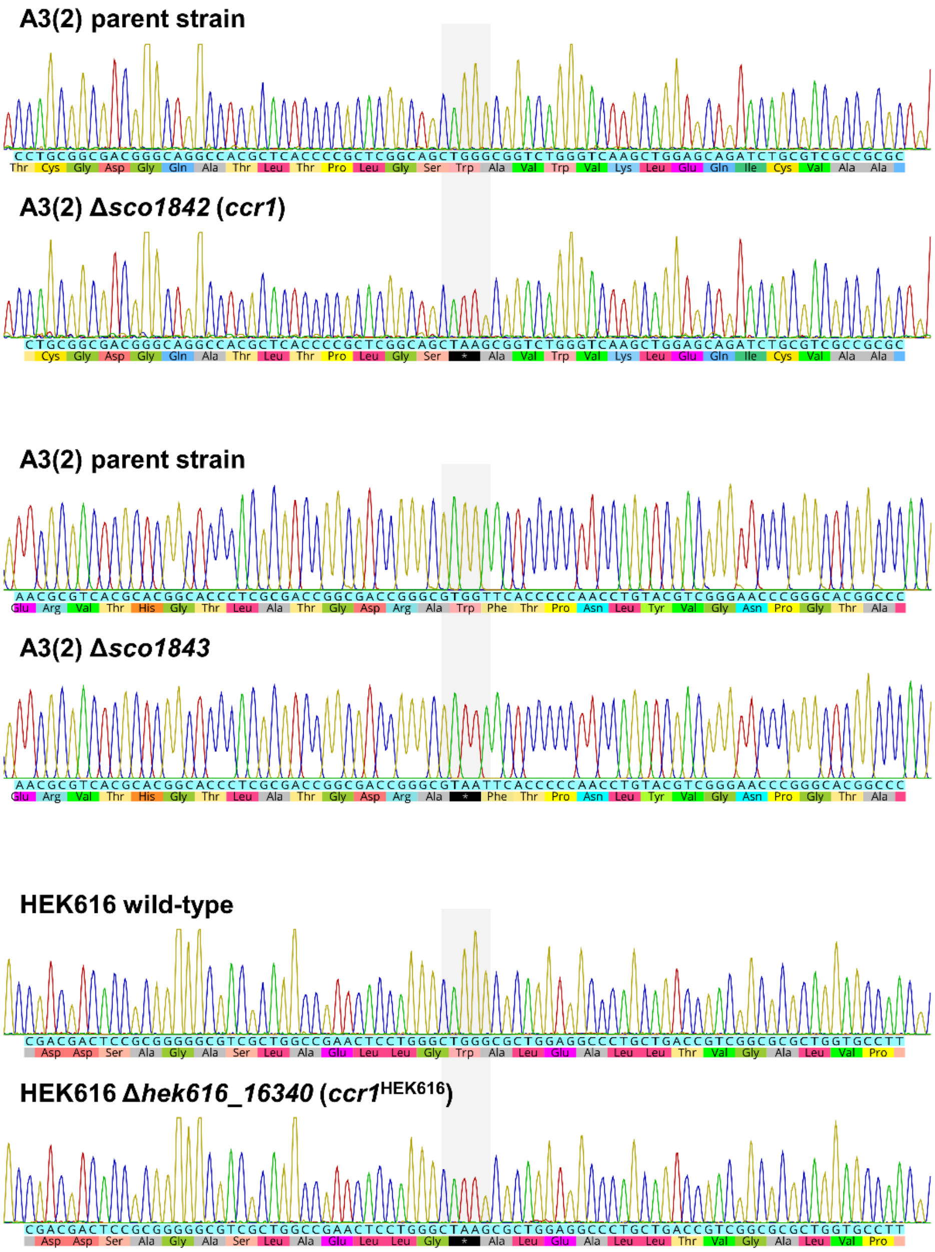

Figure S1. Sanger sequence to confirm the genome editing.

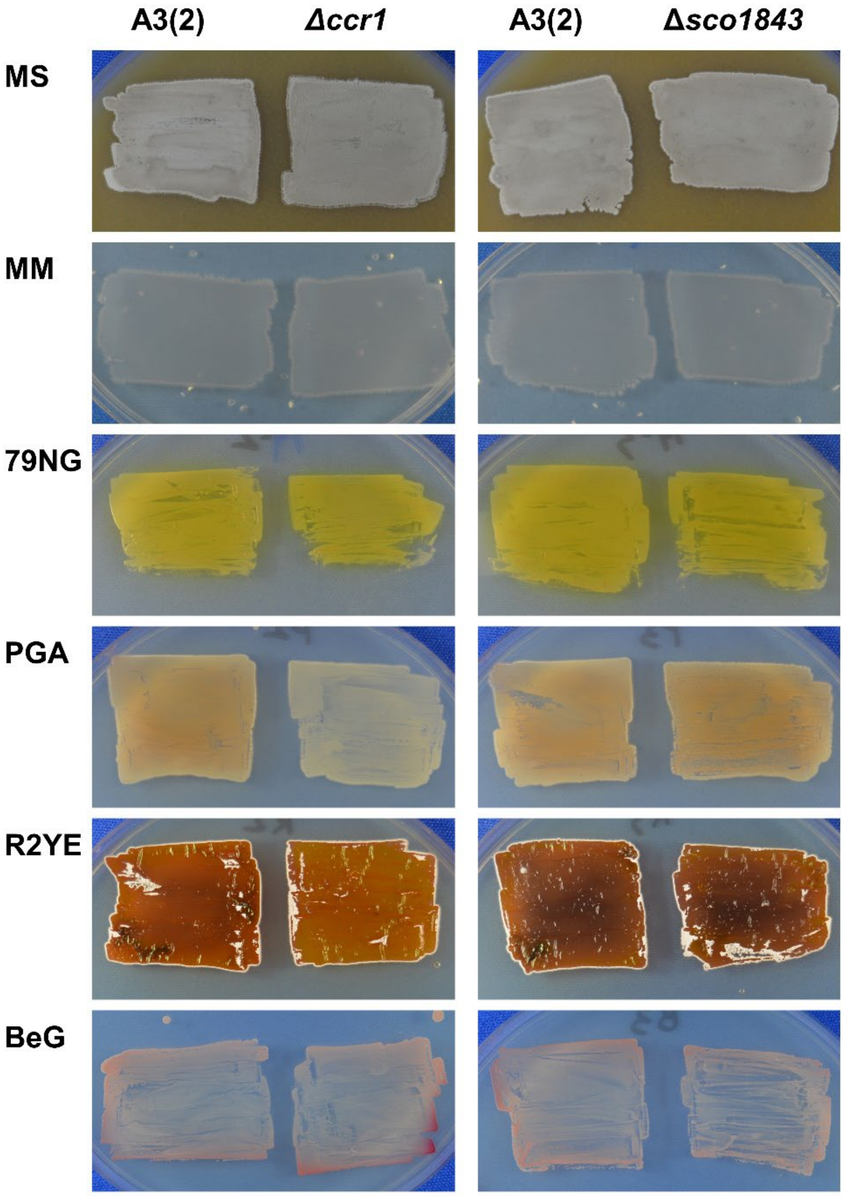

Figure S2. Phenotype of Δ*sco1842* and Δ*sco1843* mutants grown on different agar medium.

MS: mannitol-soybean medium. MM: minimal medium. BeG: Bennet's glucose medium.

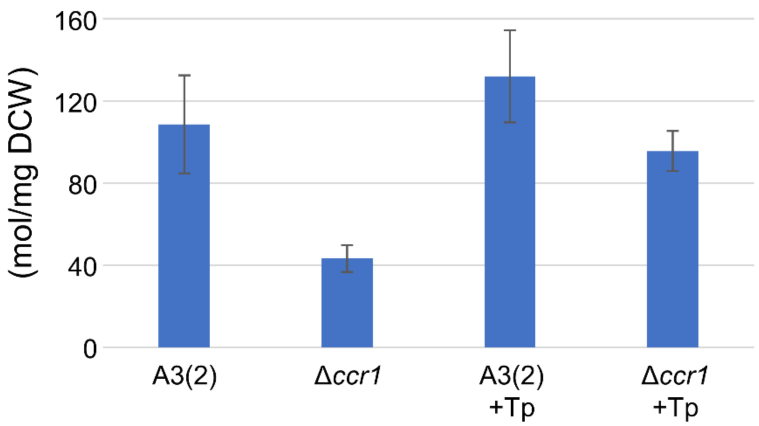

Figure S3. Quantification of RED (undecylprodigiosin).

Parent strain A3(2) and Δ*ccr1* were culture for 2 days in PGA medium.

Table S2. SCO1842 homologs with putative conserved motifs.

	Org_code	locus tag	strain name	score	e-value	conserved motif	
1	sco	SCO1842	*S. coelicolor*	857	0		
2	slv	SLIV_28505	*S. lividans*	808	0		
3	strd	NI25_30000	*Streptomyces* sp. CCM_MD2014	766	0		
4	spac	B1H29_28285	*S. pactum*	765	0		
5	spav	Spa2297_07355	*S. parvulus*	739	0		Helicase_C_3
6	samb	SAM23877_1913	*S. ambofaciens*	730	0	HTH_29/HTH_PafC	Helicase_C_3
7	scw	TU94_07810	*S. cyaneogriseus*	723	0	HTH_PafC	
8	schf	IPT68_08490	*S. chromofuscus*	715	0		
9	shaw	CEB94_09740	*S. hawaiiensis*	711	0	HTH_PafC	
10	sgu	SGLAU_08200	*S. glaucescens*	711	0	HTH_PafC	
11	saqu	EJC51_12330	*S. aquilus*	706	0	HTH_PafC	
12	sgal	CP966_07830	*S. galilaeus*	703	0		
13	scoe	CP976_10480	*S. coeruleorubidus*	703	0	HTH_PafC	
14	scya	EJ357_10560	*S. cyaneochromogenes*	702	0		
15	sphw	NFX46_29835	*S. phaeoluteigriseus*	701	0		
16	sgd	ELQ87_30355	*S. griseoviridis*	699	0		
17	stui	GCM10017668_14390	*S. tuirus*	697	0	HTH_PafC	
18	sdx	C4B68_31170	*S. dengpaensis*	695	0		BLH_phosphatase
19	srw	TUE45_02319	*S. reticuli*	697	0	HTH_PafC	
20	sjn	RI060_33095	*S. janthinus*	694	0		
21	scyg	S1361_10110	*S. cyanogenus*	691	0		
22	snz	DC008_07425	*S. nigra*	691	0	HTH_PafC	

23	sfy	GFH48_30925	*S. fagopyri*	691	0			
24	svd	CP969_10250	*S. viridosporus*	689	0	HTH_PafC		
25	sfeu	IM697_13085	*S. ferrugineus*	688	0			
26	splu	LK06_006185	*S. pluripotens*	686	0			
27	stsi	A4E84_09295	*S. qaidamensis*	685	0			
28	scad	DN051_29330	*S. cadmiisoli*	686	0			
29	sgs	AVL59_36020	*S. griseochromogenes*	684	0			
30	scha	CP983_33535	*S. chartreusis*	684	0	HTH_PafC		
31	sci	B446_09570	*S. collinus*	682	0			
32	spun	BFF78_32120	*S. fodineus*	683	0	HTH_PafC		
33	sle	sle_52910	*S. leeuwenhoekii*	680	0			
34	sarg	HKX69_26865	*S. argyrophyllae*	680	0			
35	slf	JEQ17_35760	*S. liliifuscus*	682	0			
36	scir	STRCI_001968	*S. cinnabarinus*	681	0			
37	scae	IHE65_34955	*S. caniscabiei*	681	0	HTH_PafC		
38	sbro	GQF42_12035	*S. broussonetiae*	679	0			
39	sdv	BN159_6682	*S. davaonensis*	676	0			
40	sgf	HEP81_05927	*S. griseofuscus*	673	0	HTH_PafC		
41	srj	SRO_5622	*S. rochei*	673	0	HTH_PafC		
42	sspn	LXH13_09490	*S. spinosirectus*	672	0	YAcAr		
43	sgm	GCM10017557_64160	*S. aurantiacus*	674	0			
44	spra	CP972_08355	*S. prasinus*	671	0			
45	sdd	D9753_27560	*S. dangxiongensis*	670	0	HTH_PafC		
46	sdec	L3078_10680	*S. deccanensis*	671	0	HTH_PafC		
47	sphv	F9278_09195	*S. phaeolivaceus*	665	0	HTH_PafC		
48	sseo	D0Z67_06150	*S. seoulensis*	665	0	HTH_PafC		
49	sls	SLINC_2106	*S. lincolnensis*	663	0	HTH_PafC		
50	kbu	Q4V64_11395	*Kutzneria buriramensis*	661	0	HTH_PafC		
51	sge	DWG14_06565	*S. griseorubiginosus*	661	0			
52	sho	SHJGH_3064	*S. hygroscopicus* TL01	660	0			
53	shy	SHJG_3299	*S. hygroscopicus* 5008	660	0			
54	scx	AS200_34040	*Streptomyces* sp. CdTB01	660	0			
55	sma	SAVERM_6423	*S. avermitilis*	660	0	HTH_PafC		
56	sakb	K1J60_34880	*S. akebiae*	659	0	HTH_PafC		
57	sgrf	SGFS_088420	*S. graminofaciens*	657	0	HTH_PafC		
58	salw	CP975_07780	*S. alboniger*	654	0	HTH_PafC		Helicase_C_3
59	strt	A8713_06755	*Streptomyces* sp. SAT1	647	0		DUF4589	
60	sast	CD934_26455	*S. calvus*	647	0			
61	scb	SCAB_70941	*S. scabiei*	643	0	HTH_PafC		
62	stee	F3L20_08595	*S. tendae*	639	0			
63	sfug	CNQ36_07930	*S. fungicidicus*	632	0			
64	sxn	IAG42_27590	*S. xanthii*	621	0			

65	slon	LGI35_12840	*S. longhuiensis*	619	0			Helicase_C_3
66	sspb	CP982_10915	*S. spectabilis*	618	0			
67	sals	SLNWT_6029	*S. albus*	617	0			
68	scyn	N8I84_10220	*S. cynarae*	612	0	HTH_PafC		
69	sky	D0C37_26900	*S. koyangensis*	611	0			
70	ssia	A7J05_27685	*S. alfalfae*	609	0	HTH_PafC		Helicase_C_3
71	ska	CP970_33860	*S. kanamyceticus*	609	0			
72	svio	HWN34_25135	*S. violascens*	609	0			
73	salb	XNR_4978	*S. albidoflavus*	609	0			
74	sfk	KY5_1761c	*S. formicae*	609	0			
75	sdur	M4V62_32240	*S. durmitorensis*	609	0			
76	strf	ASR50_08940	*Streptomyces* sp. 4F	605	0			
77	snq	CP978_08780	*S. nodosus*	604	0			
78	srug	F0345_05245	*S. rutgersensis*	603	0			
79	shk	J2N69_07620	*S. huasconensis*	600	0			
80	salf	SMD44_01986	*S. alboflavus*	603	0			
81	sroi	IAG44_31205	*S. roseirectus*	582	0			
82	saov	G3H79_29195	*S. aureoverticillatus*	582	0			
83	sata	C5746_10105	*S. atratus*	573	0			
84	ssx	SACTE_1269	*Streptomyces* sp. SirexAA-E	569	0			
85	speu	CGZ69_07910	*S. peucetius* subsp. *caesius*	569	0			
86	slia	HA039_26720	*S. liangshanensis*	568	0			
87	sdrz	NEH16_24875	*S. drozdowiczii*	565	0			
88	scz	ABE83_28735	*Streptomyces* sp. CFMR7	565	0			
89	snk	CP967_27105	*S. nitrosporeus*	560	0			
90	snw	BBN63_27275	*S. niveus*	559	0			
91	sgj	IAG43_06760	*S. genisteinicus*	557	0			
92	scin	CP977_08310	*S. cinereoruber*	557	0			
93	slx	SLAV_28165	*S. lavendulae* subsp. *lavendulae*	559	0			
94	svn	CP980_25540	*S. vinaceus*	557	0			
95	syan	NRK68_08330	*S. yangpuensis*	556	0			
96	svt	SVTN_08915	*S. vietnamensis*	555	0			
97	sanl	KZO11_06780	*S. anulatus*	555	0			
98	staa	LDH80_30055	*S. tanashiensis*	554	0			
99	sgb	WQO_07065	*S. globisporus*	552	0		AcetDehyd-dimer	
100	scav	CVT27_05945	*S. cavourensis*	553	0			
101	scal	I6J39_06405	*S. californicus*	551	0			
102	srk	FGW37_07835	*S. rectiverticillatus*	552	0			
103	snf	JYK04_02521	*S. nojiriensis*	552	0			
104	sve	SVEN_1489	*S. venezuelae*	551	0			

105	svu	B1H20_06235	*S. violaceoruber*	551	0			
106	sfa	Sfla_4973	*S. pratensis*	546	0			
107	strp	F750_1705	*Streptomyces* sp. PAMC26508	544	0			
108	strm	M444_09620	*Streptomyces* sp. Mg1	545	0			
109	smob	J7W19_07110	*S. mobaraensis*	545	0		HEAT_2	Helicase_C_3
110	sgz	C0216_22500	*S. globosus*	543	0			
111	sgx	H4W23_09355	*S. gardneri*	543	0			
112	sfic	EIZ62_25885	*S. ficellus*	540	0			
113	sfi	SFUL_1419	*S. microflavus*	541	0			
114	kab	B7C62_06050	*Kitasatospora albolonga*	538	0			
115	sclf	BB341_22725	*S. clavuligerus*	538	0			
116	slau	SLA_1400	*S. laurentii*	536	0			
117	srim	CP984_31735	*S. rimosus*	538	0			
118	spad	DVK44_05245	*S. paludis*	535	0			
119	sxt	KPP03845_102092	*S. xanthophaeus*	536	0			
120	sld	T261_6301	*S. lydicus*	533	0			HEAT_2
121	slk	SLUN_08900	*S. lunaelactis*	528	0			
122	sbh	SBI_08151	*S. bingchenggensis*	524	0	HTH_PafC		
123	sine	KI385_11860	*S. inhibens*	526	0			HEAT_2
124	shun	DWB77_05873	*S. hundungensis*	523	1.00E-180			
125	slc	SL103_09940	*S. lydicus* 103	523	2.00E-180			HEAT_2
126	stro	STRMOE7_10155	*Streptomyces* sp. MOE7	522	5.00E-180			HEAT_2
127	spri	SPRI_5678	*S. pristinaespiralis*	520	1.00E-179			
128	sfb	CP974_05570	*S. fradiae*	517	5.00E-178			
129	ssub	CP968_25350	*S. subrutilus*	518	6.00E-178			Helicase_C_3
130	stir	DDW44_07705	*S. tirandamycinicus*	517	2.00E-177			
131	salj	SMD11_5150	*S. albireticuli*	514	2.00E-177	HTH_28		

132	sqz	FQU76_05935	*S. qinzhouensis*	514	7.00E-177		Helicase_C_3
133	snr	SNOUR_30995	*S. noursei* ATCC11455	512	5.00E-176		HEAT_2
134	sdw	K7C20_08765	*S. decoyicus*	513	6.00E-176	HTH_PafC/Xre-like-HTH/HTH_28	HEAT_2
135	sanu	K7396_27565	*S. angustmyceticus*	511	3.00E-175	HTH_PafC	HEAT_2
136	salu	DC74_2348	*S. noursei* NK660	509	4.00E-174	HTH_28	HEAT_2
137	sgv	B1H19_11325	*S. gilvosporeus*	505	1.00E-172		HEAT_2
138	syun	MOV08_31015	*S. yunnanensis*	504	2.00E-172		
139	stre	GZL_06661	*Streptomyces* sp. 769	503	2.00E-172		HEAT_2
140	spla	CP981_09255	*S. platensis*	501	1.00E-171		HEAT_2
141	src	M271_37025	*S. rapamycinicus*	501	2.00E-171		
142	sauo	BV401_12495	*S. autolyticus*	499	1.00E-170		
143	ssoi	I1A49_12180	*S. solisilvae*	499	1.00E-170		
144	smal	SMALA_1806	*S. malaysiensis*	499	1.00E-170		
145	snig	HEK616_16340	*S. nigrescens*	496	4.00E-169		HEAT_2
146	srn	A4G23_01072	*S. rubrolavendulae*	493	4.00E-169		
147	sgob	test1122_02310	*S. gobiensis*	494	1.00E-168		
148	stub	MMF93_07225	*S. tubbatahanensis*	492	1.00E-168		
149	stsu	B7R87_05925	*S. tsukubensis*	494	1.00E-168		
150	sby	H7H31_28055	*S. buecherae*	495	2.00E-168		Helicase_C_3
151	stud	STRTU_005535	*S. tubercidicus*	493	5.00E-168		HEAT_2
152	svl	Strvi_6933	*S. violaceusniger*	483	3.00E-164		
153	sauh	SU9_007240	*S. auratus*	481	1.00E-163		HEAT_2
154	sant	QR300_32615	*S. antimycoticus*	481	1.00E-163		
155	sbat	G4Z16_27330	*S. bathyalis*	480	5.00E-163		
156	strc	AA958_05260	*Streptomyces* sp. CNQ-509	463	4.00E-157		HEAT_2
157	scy	SCATT_10330	*Streptantibioticus cattleyicolor* NRRL 8057	463	5.00E-157		

158	sct	SCAT_1040	*Streptantibioticus cattleyicolor* NRRL 8057	463	5.00E-157		
159	abry	NYE86_10295	*Actinacidiphila bryophytorum*	458	9.00E-155		
160	smao	CAG99_23610	*S. marincola*	430	1.00E-143	DnaG_DnaB_bind	
161	sxi	SXIM_08260	*S. xiamenensis*	422	1.00E-140		
162	shar	HUT13_04690	*S. harbinensis*	406	2.00E-134		Transglut_core
163	sgr	SGR_5650	*S. griseus*	394	1.00E-129		
164	sspo	DDQ41_05670	*S. spongiicola*	363	3.00E-116		
165	kis	HUT16_26300	*Kitasatospora* sp. NA04385	327	5.00E-104		Transgly
166	ksk	KSE_56440	*Kitasatospora setae*	327	7.00E-104		Transgly
167	strh	GXP74_30700	*Streptacidiphilus* sp. P02-A3a	325	2.00E-103		
168	kit	CFP65_5567	*Kitasatospora* sp. MMS16-BH015	322	4.00E-102		
169	stri	C7M71_023975	*Peterkaempfera bronchialis*	320	5.00E-101		
170	sall	SAZ_13090	*S. noursei* ZPM	112	1.00E-25		
171	actw	F7P10_36715	*Actinomadura* sp. WMMB 499	100	1.00E-19		

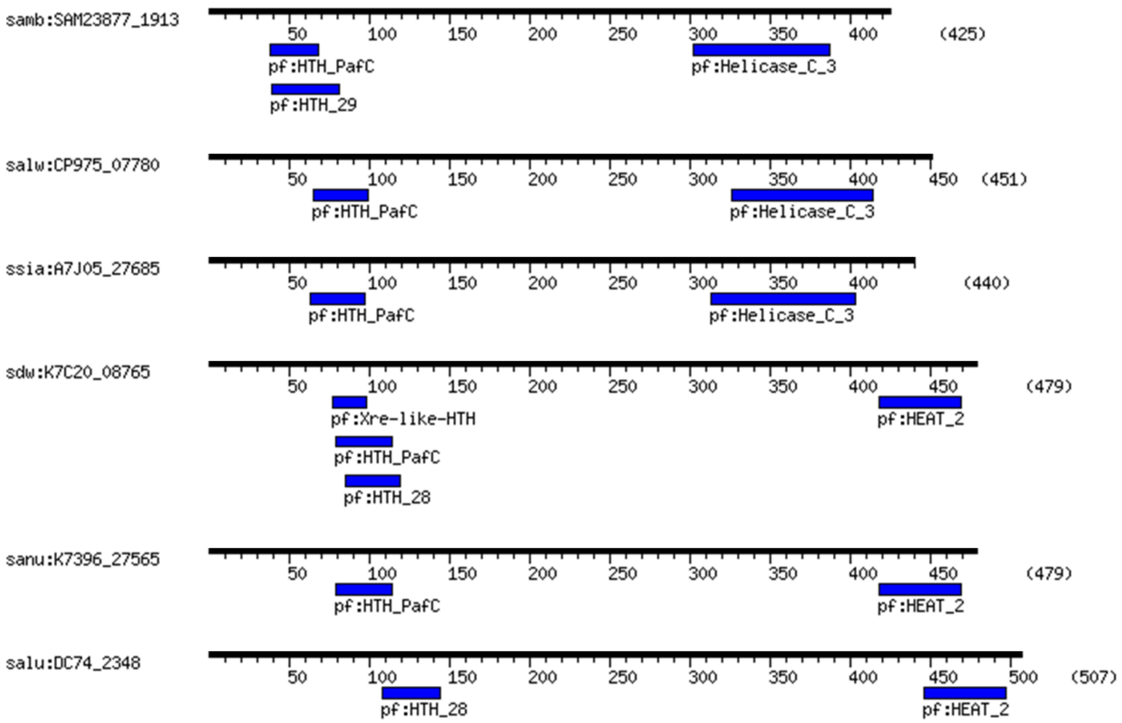

Figure S4. Predicted motifs of SCO1842 homologs found in the KEGG database.

SCO1842 homologs containing HTH/HCTD (Helicase C-terminal Domain) (A7J05_27685, CP975_07780, SAM23877_1913) motif or HTH/HEAT (DC74_2348, K7396_27565, K7C20_08765) motif were shown.

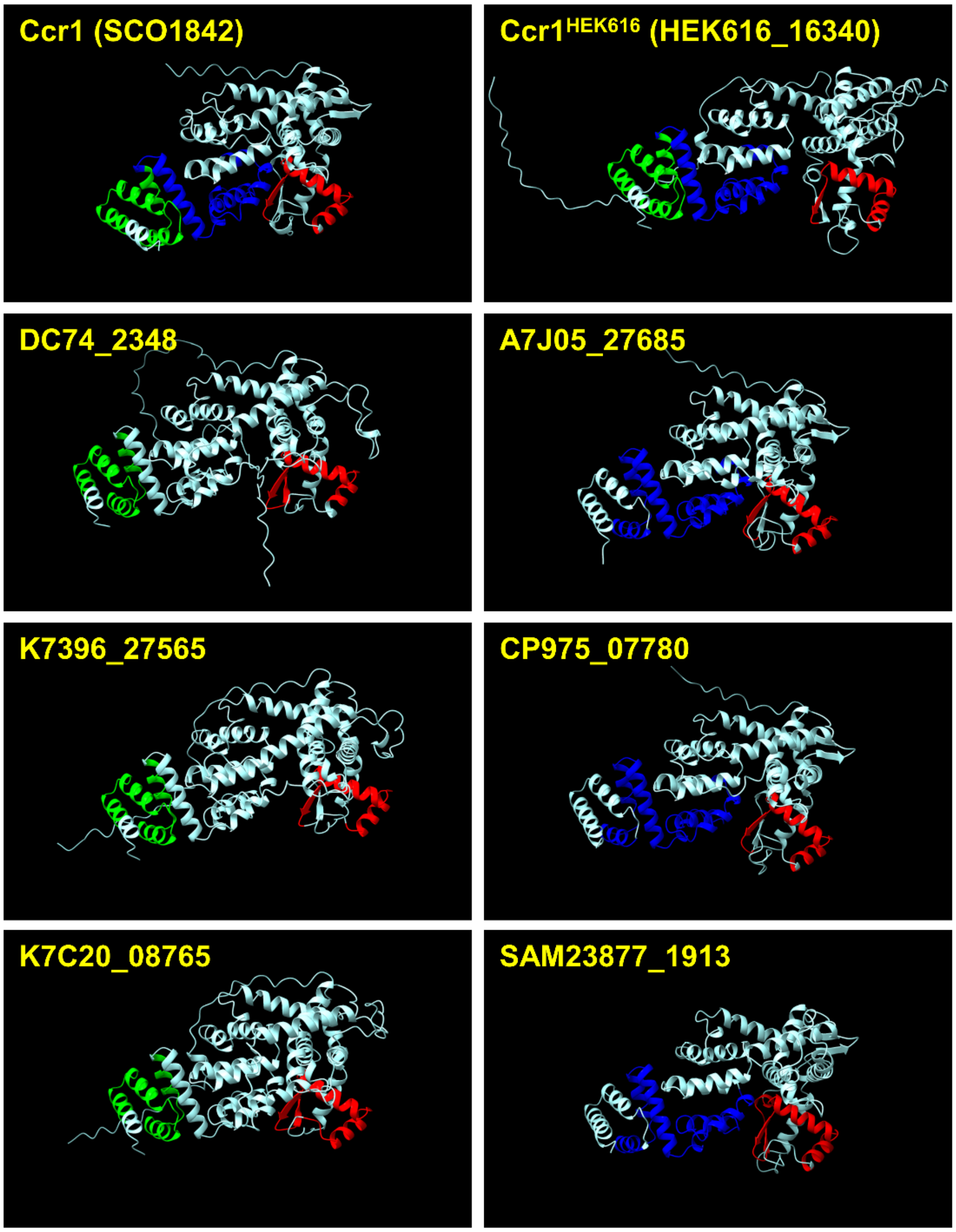

Figure S5. Predicted 3D structure of SCO1842 homologs by AlfaFold2 (ColabFold).

3D structure of SCO1842 homologs containing HTH/HCTD (A7J05_27685, CP975_07780, SAM23877_1913) motif or HTH/HEAT (DC74_2348, K7396_27565, K7C20_08765) motif were predicted. Red color indicates the position of putative HTH motif. Green color indicates the putative HEAT motif. Blue color indicates the putative HCTD motif. Predicted positions of HTH, HCTD and HEAT motif in SCO1842 and HEK616_16340 are colored. (SCO1842: pLDDT=88.4, pTM=0.855; HEK616_16340: pLDDT=81.4, pTM=0.651; DC74_2348: pLDDT=80.6, pTM=0.694; K7396_27565: pLDDT=84.4, pTM=0.76; K7C20_08765: pLDDT=83.6, pTM=0.798; A7J05_27685: pLDDT=88.8, pTM=0.866; CP975_07780: pLDDT=87, pTM=0.737; SAM23877: pLDDT=87.8, pTM=0.745)

Table S3. List of differentially expressed genes in the RNA-seq analysis of Δ*sco1842*.

(You can find in an additional supplemental data).

Our detailed RNA-seq analysis revealed that the conservon region (*cvn13* genes) exhibited up-regulation in Δ*ccr1* (Table S3). In another bacterial interaction study, Bonet et al. demonstrated up-regulation of a conservon region (*cvn8*) that resulted from interaction with *Amicolatopsis* sp. AA4 (Bonet et al., 2021). *cvn13* expression was reported to be repressed upon ppGpp synthesis in *S. coelicolor* M653 [Δ*relA*, *tipAp::relA*] (Hesketh et al., 2007), which may indicate the depletion of cellular ppGpp in Δ*ccr1*. ppGpp synthesis is an important trigger for RED production, which is likely consistent with our observation. Furthermore, the ECF sigma factor SCO4005, which is up-regulated upon ppGpp synthesis, was highly repressed in Δ*ccr1*. This indicates that ppGpp may be involved in the regulatory role of Ccr1.

There are some other possible regulatory roles of Ccr1 deduced from our RNA-seq analysis, such as ribosome constitution and translation. This can be inferred by the transcription level change of an elongation factor G (EF-G) gene, *sco1528*, and several ribosomal protein genes including *rpsL33* (*sco0570*), *rpsL25* (*sco3124*), *rpsL10* (*sco4701*), and *rpsL4* (*sco4703*). Interestingly, we previously identified another EF-G gene, *fusA* (*sco4661*), as responsible for reduced RED production (Yanagisawa et al., 2022). It is known that mutations in some ribosomal proteins can affect translation and thus secondary metabolism (Okamoto-Hosoya et al., 2003; Lopatniuk et al., 2019); therefore, the effect of Ccr1 on secondary metabolism may also be partly exerted by impacting protein translation.

As previously mentioned, there was significant down-regulation of *devR* (0.26-fold, data not shown) and its regulon (*sco0162-81*) in Δ*ccr1*. DevR is a response regulator (RR) of the two-component system (TCS) DevS/R (Urem et al., 2016). DevR directly regulates ACT production in *S. coelicolor* A3(2) (Urem et al., 2016). This regulation relies on the signaling molecule nitrite oxide (NO) (Honma et al., 2021), which is endogenously generated by three nonredundant Nar enzymes, Nar1 (SCO6532-35), Nar2 (SCO0216-19), and Nar3 (SCO4947-50) (Fischer et al., 2010). Knockout of these Nar enzymes results in a lack of NO production and, as a result, a loss of ACT production (Fischer et al., 2014). Interestingly, Nar2 and Nar3 expression levels were reduced by 0.83 to 0.71-fold (data not shown) and 0.2 to 0.11-fold, respectively, in Δ*ccr1*, which the phenotypes are consistent with the findings of the previous studies. It is also possible that *ccr1* knockout may lead to the down-regulation of NO production and therefore the DevS/R system, resulting in significant down-regulation of ACT biosynthetic genes.

We also found that *btdA* (*sco3328*) was down-regulated in the Δ*ccr1* strain. Although the function of *btdA* remains unclear, TCS-RR AbrC3 is known to positively regulate *btdA* (Rico et al., 2014). AbrC3 inactivation resulted in reduced ACT and RED production, which caused a delay in morphological development (Rico et al., 2014). Collectively, these results indicate that the function of AbrC3 was repressed in the Δ*ccr1* strain, which was consistent with the AbrC3 knockout mutant phenotype. Additionally, *btdA* is also a known target of BldD (den Hengst et al., 2010). During the vegetative growth phase, BldD forms a complex with c-di-GMP and represses genes associated with morphological development (Tschowri et al., 2014). Although *btdA* inactivation showed no apparent effect on growth and its function remains unclear (den

Hengst et al., 2010), it is possible that the Δ*ccr1* strain retains a higher cellular concentration of c-di-GMP.

This is also supported by the finding that *cdgC* (*sco5511*), a gene responsible for controlling cellular levels of c-di-GMP (Latoscha et al., 2019), was down-regulated in the Δ*ccr1* strain. CdgC is a membrane-bound protein that contains PAS/PAC sensor domain, cyclase (c-di-GMP synthesis) domain, and hydrolase (c-di-GMP hydrolysis) domain (Latoscha et al., 2019). *cdgC* activation did not visibly affect morphology (Liu et al., 2019). Because overexpression of the diguanylate cyclase domain of CdgC led to increased ACT production (Liu et al., 2019), excess accumulation of c-di-GMP may disturb cellular processes. Retention of a cellular concentration of c-di-GMP was predicted, and both synthesis and hydrolysis activity by CdgC may be diminished in Δ*ccr1* by down-regulation of *cdgC*.

Furthermore, down-regulation of *ssgB* (*sco1521*), whose product is involved in recruiting FtsZ for cell division (Willemse et al., 2011), was observed in the Δ*ccr1* strain. The ECF σ-factor SigH (SCO5243) is essential for *ssgB* expression (Kormanec and Sevcikova, 2002). *sigH* is induced after osmotic stress and heat shock, and plays a crucial role in morphological development (Sevcikova et al., 2001). Therefore, stress response involving SigH may not be associated with the Ccr1 regulon. Nevertheless, although the impact on SM production was significant, our RNA-seq analysis suggests the possibility that Ccr1 may also influence morphological development that was not immediately apparent by observing the colony's growth on an agar plate.

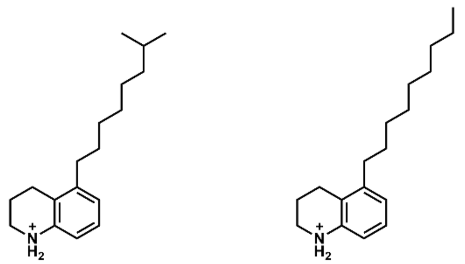

5aTHQ-9i 5aTHQ-9n

Chemical Formula: $C_{18}H_{30}N^+$
Exact Mass: 260.2373 (calc.)
m/z: 260.2373 (100.0%), 261.2406 (19.5%), 262.2440 (1.8%)

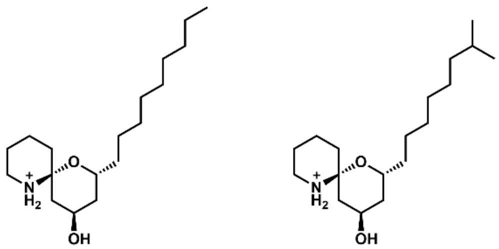

Streptoaminal-9i Streptoaminal-9n

Chemical Formula: $C_{18}H_{36}NO_2^+$
Exact Mass: 298.2741 (calc.)
m/z: 298.2741 (100.0%), 299.2774 (19.5%), 300.2808 (1.8%)

Figure S6. Chemical structures of streptoaminals (9i, 9n), and 5aTHQ (9i, 9n).

The *m/z* values of the isotopes (261.2406 ± 20 ppm for 5aTHQs and 299.2774 ± 20 ppm for streptoaminals) were used to draw the extracted ion chromatograph (EIC) since peak of monoisotopic ion exceeded saturation level of the dynamic range.

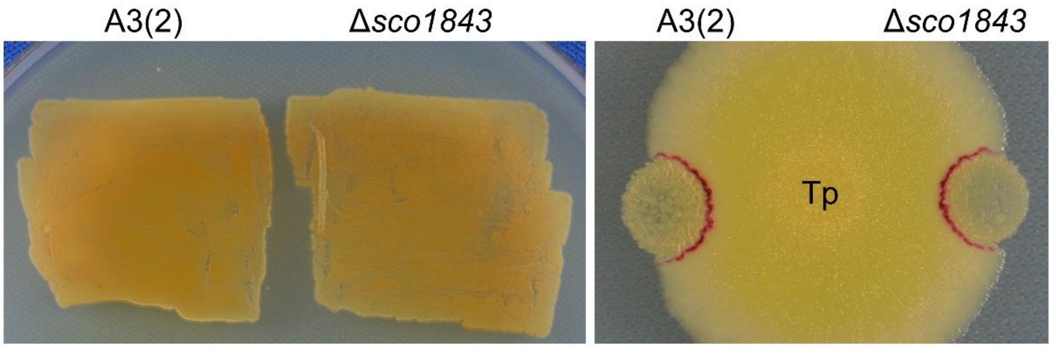

Figure S7. Impact of *sco1843* on RED production.

RED production was compared between mono-culture (day 2) performed in PGA medium and competitive cultures (day 4) performed in YGGS medium.

Table S4. RT-qPCR analysis of *sco1842* and genes in flanking region.

	mono-culture		combined-culture		
gene	nor_ave	stdev	nor_ave	stdev	fold change
sco1845	44.89	4.99	7.55	3.66	0.17
sco1844	9.05	2.36	8.63	2.37	0.95
sco1843	2.38	0.82	63.88	3.69	26.84
sco1842	5.45	1.39	33.86	3.62	6.21
sco1841	24.80	7.52	73.75	1.13	2.97
sco1840	67.53	11.01	44.72	4.53	0.66

Table S5. Conservation of SCO1843 homologs.

	org _code	SCO1842 homologs	SCO1843 homologs	score (SCO1843)	e-value (SCO1843)
1	sco	SCO1842	SCO1843	718	0
2	slv	SLIV_28505	SLIV_28500	718	0
3	strd	NI25_30000	NI25_29995	649	0
4	spac	B1H29_28285	B1H29_28280	625	0
5	spav	Spa2297_07355	Spa2297_07360	632	0
6	samb	SAM23877_1913	SAM23877_1914	633	0
7	scw	TU94_07810	TU94_07815	528	0
8	schf	IPT68_08490	IPT68_08495	473	3.00E-163
9	shaw	CEB94_09740	CEB94_09745	508	1.00E-176
10	sgu	SGLAU_08200	SGLAU_08205	483	5.00E-167
11	saqu	EJC51_12330	EJC51_12335	496	3.00E-172
12	sgal	CP966_07830	CP966_07835	491	4.00E-170
13	scoe	CP976_10480	CP976_10485	518	1.00E-180
14	scya	EJ357_10560	EJ357_10565	511	5.00E-178
15	sphw	NFX46_29835	NFX46_29840	491	2.00E-170
16	sgd	ELQ87_30355	ELQ87_30350	442	5.00E-151
17	stui	GCM10017668_14390	GCM10017668_14400	511	5.00E-178
18	sdx	C4B68_31170	C4B68_31165	478	2.00E-164
19	srw	TUE45_02319	TUE45_02320	504	9.00E-175
20	sjn	RI060_33095	RI060_33090	509	4.00E-177
21	scyg	S1361_10110	S1361_10115	487	1.00E-167
22	snz	DC008_07425	DC008_07430	525	0
23	sfy	GFH48_30925	GFH48_30920	485	7.00E-168
24	svd	CP969_10250	CP969_10255	476	3.00E-164
25	sfeu	IM697_13085	IM697_13080	508	1.00E-176
26	splu	LK06_006185	LK06_006190	531	0
27	stsi	A4E84_09295	A4E84_09300	511	5.00E-178
28	scad	DN051_29330	DN051_29325	459	2.00E-157
29	sgs	AVL59_36020	AVL59_36025	499	2.00E-173
30	scha	CP983_33535	CP983_33530	491	2.00E-170
31	sci	B446_09570	B446_09575	507	1.00E-175
32	spun	BFF78_32120	BFF78_32115	516	7.00E-180
33	sle	sle_52910	sle_52900	516	8.00E-180
34	sarg	HKX69_26865	HKX69_26860	518	1.00E-180
35	slf	JEQ17_35760	JEQ17_35755	508	1.00E-176
36	scir	STRCI_001968	STRCI_001969	490	8.00E-170
37	scae	IHE65_34955	IHE65_34950	437	6.00E-149
38	sbro	GQF42_12035	GQF42_12040	499	1.00E-173
39	sdv	BN159_6682	BN159_6681	496	4.00E-172
40	sgf	HEP81_05927	HEP81_05926	501	3.00E-173
41	srj	SRO_5622	SRO_5621	501	3.00E-173
42	sspn	LXH13_09490	LXH13_09495	503	9.00E-175
43	sgm	GCM10017557_64160	GCM10017557_64150	467	1.00E-160
44	spra	CP972_08355	CP972_08360	478	4.00E-165

45	sdd	D9753_27560	D9753_27555	503	6.00E-175
46	sdec	L3078_10680	L3078_10685	441	2.00E-150
47	sphv	F9278_09195	F9278_09200	436	3.00E-148
48	sseo	D0Z67_06150	D0Z67_06155	464	2.00E-159
49	sls	SLINC_2106	SLINC_2107	497	2.00E-172
50	kbu	Q4V64_11395	Q4V64_11405	500	9.00E-174
51	sge	DWG14_06565	DWG14_06564	480	2.00E-165
52	sho	SHJGH_3064	SHJGH_3065	538	0
53	shy	SHJG_3299	SHJG_3300	538	0
54	scx	AS200_34040	AS200_34035	487	1.00E-168
55	sma	SAVERM_6423	SAVERM_6422	507	2.00E-176
56	sakb	K1J60_34880	K1J60_34875	440	6.00E-150
57	sgrf	SGFS_088420	SGFS_088410	471	3.00E-162
58	salw	CP975_07780	CP975_07785	459	2.00E-157
59	strt	A8713_06755	A8713_06760	493	6.00E-171
60	sast	CD934_26455	CD934_26450	466	4.00E-160
61	scb	SCAB_70941	SCAB_70931	432	5.00E-147
62	stee	F3L20_08595	F3L20_08590	468	7.00E-161
63	sfug	CNQ36_07930	CNQ36_07935	455	6.00E-156
64	sxn	IAG42_27590	IAG42_27585	485	8.00E-168
65	slon	LGI35_12840	LGI35_12845	447	1.00E-152
66	sspb	CP982_10915	CP982_10920	434	8.00E-148
67	sals	SLNWT_6029	SLNWT_6028	423	3.00E-143
68	scyn	N8I84_10220			
69	sky	D0C37_26900	D0C37_26895	416	1.00E-140
70	ssia	A7J05_27685	A7J05_27680	431	2.00E-146
71	ska	CP970_33860	CP970_33855	461	2.00E-158
72	svio	HWN34_25135	HWN34_25130	417	5.00E-141
73	salb	XNR_4978	XNR_4977	417	5.00E-141
74	sfk	KY5_1761c	KY5_1762c	475	8.00E-164
75	sdur	M4V62_32240	M4V62_32235	475	6.00E-164
76	strf	ASR50_08940	ASR50_08945	469	2.00E-161
77	snq	CP978_08780	CP978_08785	448	4.00E-153
78	srug	F0345_05245	F0345_05255	423	2.00E-143
79	shk	J2N69_07620	J2N69_07625	444	1.00E-151
80	salf	SMD44_01986	SMD44_01987	476	2.00E-164
81	sroi	IAG44_31205	IAG44_31200	458	3.00E-157
82	saov	G3H79_29195	G3H79_29190	479	3.00E-165
83	sata	C5746_10105	C5746_10110	425	4.00E-144
84	ssx	SACTE_1269	SACTE_1270	418	2.00E-141
85	speu	CGZ69_07910	CGZ69_07915	414	7.00E-140
86	slia	HA039_26720	HA039_26715	440	5.00E-150
87	sdrz	NEH16_24875			
88	scz	ABE83_28735	ABE83_28730	419	1.00E-141
89	snk	CP967_27105	CP967_27100	421	1.00E-142
90	snw	BBN63_27275	BBN63_27270	438	2.00E-149
91	sgj	IAG43_06760	IAG43_06765	408	1.00E-137
92	scin	CP977_08310	CP977_08315	411	8.00E-139
93	slx	SLAV_28165	SLAV_28160	388	2.00E-129
94	svn	CP980_25540	CP980_25535	412	5.00E-139
95	syan	NRK68_08330	NRK68_08335	419	9.00E-142
96	svt	SVTN_08915	SVTN_08920	412	2.00E-139
97	sanl	KZO11_06780	KZO11_06785	416	7.00E-141
98	staa	LDH80_30055	LDH80_30050	432	7.00E-147
99	sgb	WQO_07065	WQO_07070	429	8.00E-146
100	scav	CVT27_05945	CVT27_05950	419	9.00E-142
101	scal	I6J39_06405	I6J39_06410	411	9.00E-139
102	srk	FGW37_07835	FGW37_07840	384	7.00E-128
103	snf	JYK04_02521	JYK04_02522	407	5.00E-137
104	sve	SVEN_1489	SVEN_1490	416	2.00E-140
105	svu	B1H20_06235	B1H20_06240	408	2.00E-137
106	sfa	Sfla_4973	Sfla_4972	410	4.00E-138
107	strp	F750_1705	F750_1706	412	6.00E-139
108	strm	M444_09620	M444_09625	399	4.00E-134
109	smob	J7W19_07110	J7W19_07115	275	3.00E-85
110	sgz	C0216_22500	C0216_22505	393	9.00E-132
111	sgx	H4W23_09355	H4W23_09360	404	6.00E-136
112	sfic	EIZ62_25885	EIZ62_25880	427	6.00E-145

113	sfi	SFUL_1419	SFUL_1420	421	2.00E-142
114	kab	B7C62_06050	B7C62_06055	400	2.00E-134
115	sclf	BB341_22725	BB341_22720	409	1.00E-137
116	slau	SLA_1400	SLA_1401	423	9.00E-144
117	srim	CP984_31735	CP984_31730	426	1.00E-144
118	spad	DVK44_05245	DVK44_05250	429	1.00E-145
119	sxt	KPP03845_102092	KPP03845_102093	420	5.00E-142
120	sld	T261_6301	T261_6300	385	2.00E-128
121	slk	SLUN_08900	SLUN_08905	433	2.00E-147
122	sbh	SBI_08151	SBI_08150	426	2.00E-144
123	sine	KI385_11860	KI385_11865	399	6.00E-134
124	shun	DWB77_05873	DWB77_05872	464	5.00E-160
125	slc	SL103_09940	SL103_09935	399	6.00E-134
126	stro	STRMOE7_10155	STRMOE7_10160	398	1.00E-133
127	spri	SPRI_5678	SPRI_5677	412	5.00E-139
128	sfb	CP974_05570	CP974_05575	395	3.00E-132
129	ssub	CP968_25350	CP968_25345	387	2.00E-129
130	stir	DDW44_07705	DDW44_07710	434	8.00E-148
131	salj	SMD11_5150	SMD11_5149	395	2.00E-132
132	sqz	FQU76_05935	FQU76_05940	382	2.00E-127
133	snr	SNOUR_30995	SNOUR_30990	391	7.00E-131
134	sdw	K7C20_08765	K7C20_08770	387	3.00E-129
135	sanu	K7396_27565	K7396_27560	387	4.00E-129
136	salu	DC74_2348	DC74_2349	395	1.00E-132
137	sgv	B1H19_11325	B1H19_11330	409	5.00E-138
138	syun	MOV08_31015	MOV08_31010	392	2.00E-131
139	stre	GZL_06661	GZL_06660	393	1.00E-131
140	spla	CP981_09255	CP981_09260	386	7.00E-129
141	src	M271_37025	M271_37020	410	2.00E-138
142	sauo	BV401_12495	BV401_12500	407	5.00E-137
143	ssoi	I1A49_12180	I1A49_12185	405	1.00E-136
144	smal	SMALA_1806	SMALA_1807	406	1.00E-136
145	snig	HEK616_16340	HEK616_16330	380	9.00E-127
146	srn	A4G23_01072	A4G23_01073	400	1.00E-134
147	sgob	test1122_02310	test1122_02315	379	8.00E-126
148	stub	MMF93_07225	MMF93_07230	367	2.00E-121
149	stsu	B7R87_05925	B7R87_05930	387	3.00E-129
150	sby	H7H31_28055	H7H31_28050	437	5.00E-149
151	stud	STRTU_005535	STRTU_005534	387	2.00E-129
152	svl	Strvi_6933	Strvi_6934	399	5.00E-134
153	sauh	SU9_007240	SU9_007245	399	4.00E-134
154	sant	QR300_32615	QR300_32610	394	7.00E-132
155	sbat	G4Z16_27330	G4Z16_27325	325	1.00E-104
156	strc	AA958_05260	AA958_05265	270	1.00E-83
157	scy	SCATT_10330	SCATT_10340	358	4.00E-118
158	sct	SCAT_1040	SCAT_1041	358	4.00E-118
159	abry	NYE86_10295	NYE86_10290	349	2.00E-114
160	smao	CAG99_23610	CAG99_23605	323	2.00E-104
161	sxi	SXIM_08260	SXIM_08270	285	3.00E-89
162	shar	HUT13_04690	HUT13_04695	280	1.00E-87
163	sgr	SGR_5650	SGR_5649	412	6.00E-139
164	sspo	DDQ41_05670	DDQ41_05675	394	3.00E-132
165	kis	HUT16_26300	HUT16_26295	277	1.00E-86
166	ksk	KSE_56440	KSE_56430	274	8.00E-85
167	strh	GXP74_30700	GXP74_30705	270	9.00E-84
168	kit	CFP65_5567	CFP65_5566	282	2.00E-88
169	stri	C7M71_023975	C7M71_023965	308	2.00E-98

Table S6. *Streptomyces* strains that do not possess SCO1842 or SCO1843 homologs.

org_code	strain name	SCO1842 homolog	SCO1843 homolog	score (SCO1843)	e-value (SCO1843)
stry	*Streptomyces* sp. S6	NA	NA		
seng	*S. endophytica*	NA	NA		
sact	*S. actuosus*	NA	DMT42_08430	513	5.00E-179
sgk	*S. goshikiensis*	NA	PET44_08230	399	4.00E-134
sall	*S. noursei* ZPM	NA	SAZ_13095	395	1.00E-132
svr	*S. viridifaciens*	NA	CP971_08970	243	3.00E-73

References

Asamizu, S., Pramana, A.A.C., Kawai, S.J., Arakawa, Y., and Onaka, H. (**2022**). Comparative Metabolomics Reveals a Bifunctional Antibacterial Conjugate from Combined-Culture of *Streptomyces hygroscopicus* HOK021 and *Tsukamurella pulmonis* TP-B0596. ***ACS Chem Biol*** 17(9)**,** 2664-2672. doi: 10.1021/acschembio.2c00585.

Bonet, B., Ra, Y., Cantu Morin, L.M., Soto Bustos, J., Livny, J., and Traxler, M.F. (2021). The cvn8 Conservon System Is a Global Regulator of Specialized Metabolism in Streptomyces coelicolor during Interspecies Interactions. *mSystems* 6(5)**,** e0028121. doi: 10.1128/mSystems.00281-21.

den Hengst, C.D., Tran, N.T., Bibb, M.J., Chandra, G., Leskiw, B.K., and Buttner, M.J. (2010). Genes essential for morphological development and antibiotic production in Streptomyces coelicolor are targets of BldD during vegetative growth. *Mol Microbiol* 78(2)**,** 361-379. doi: 10.1111/j.1365-2958.2010.07338.x.

Fischer, M., Alderson, J., van Keulen, G., White, J., and Sawers, R.G. (2010). The obligate aerobe Streptomyces coelicolor A3(2) synthesizes three active respiratory nitrate reductases. *Microbiology (Reading)* 156(Pt 10)**,** 3166-3179. doi: 10.1099/mic.0.042572-0.

Fischer, M., Falke, D., Pawlik, T., and Sawers, R.G. (2014). Oxygen-dependent control of respiratory nitrate reduction in mycelium of Streptomyces coelicolor A3(2). *J Bacteriol* 196(23)**,** 4152-4162. doi: 10.1128/JB.02202-14.

Hagihara, R., Katsuyama, Y., Sugai, Y., Onaka, H., and Ohnishi, Y. (**2018**). Novel desferrioxamine derivatives synthesized using the secondary metabolism-specific nitrous acid biosynthetic pathway in *Streptomyces davawensis*. ***J Antibiot (Tokyo)*** 71(11)**,** 911-919. doi: 10.1038/s41429-018-0088-1.

Hesketh, A., Chen, W.J., Ryding, J., Chang, S., and Bibb, M. (2007). The global role of ppGpp synthesis in morphological differentiation and antibiotic production in Streptomyces coelicolor A3(2). *Genome Biol* 8(8)**,** R161. doi: 10.1186/gb-2007-8-8-r161.

Honma, S., Ito, S., Yajima, S., and Sasaki, Y. (2021). Nitric Oxide Signaling for Actinorhodin Production in Streptomyces coelicolor A3(2) via the DevS/R Two-Component System. *Appl Environ Microbiol* 87(14)**,** e0048021. doi: 10.1128/AEM.00480-21.

Hoshino, S., Okada, M., Awakawa, T., Asamizu, S., Onaka, H., and Abe, I. (**2017**). Mycolic Acid Containing Bacterium Stimulates Tandem Cyclization of Polyene Macrolactam in a Lake Sediment Derived Rare Actinomycete. ***Org Lett*** 19(18)**,** 4992-4995. doi: 10.1021/acs.orglett.7b02508.

Hoshino, S., Okada, M., Wakimoto, T., Zhang, H., Hayashi, F., Onaka, H., et al. (**2015a**). Niizalactams A-C, Multicyclic Macrolactams Isolated from Combined Culture of *Streptomyces* with Mycolic Acid-Containing Bacterium. ***J Nat Prod*** 78(12)**,** 3011-3017. doi: 10.1021/acs.jnatprod.5b00804.

Hoshino, S., Ozeki, M., Awakawa, T., Morita, H., Onaka, H., and Abe, I. (**2018a**). Catenulobactins A and B, Heterocyclic Peptides from Culturing *Catenuloplanes* sp. with a Mycolic Acid-Containing Bacterium. ***J Nat Prod*** 81(9)**,** 2106-2110. doi: 10.1021/acs.jnatprod.8b00261.

Hoshino, S., Ozeki, M., Wong, C.P., Zhang, H., Hayashi, F., Awakawa, T., et al. (**2018b**). Mirilactams C-E, Novel Polycyclic Macrolactams Isolated from Combined-Culture of *Actinosynnema mirum* NBRC 14064 and Mycolic Acid-Containing Bacterium. ***Chem Pharm Bull (Tokyo)*** 66(6)**,** 660-667. doi: 10.1248/cpb.c18-00143.

Hoshino, S., Wakimoto, T., Onaka, H., and Abe, I. (**2015b**). Chojalactones A-C, cytotoxic butanolides isolated from *Streptomyces* sp. cultivated with mycolic acid containing bacterium. ***Org Lett*** 17(6), 1501-1504. doi: 10.1021/acs.orglett.5b00385.

Hoshino, S., Wong, C.P., Ozeki, M., Zhang, H., Hayashi, F., Awakawa, T., et al. (**2018c**). Umezawamides, new bioactive polycyclic tetramate macrolactams isolated from a combined-culture of *Umezawaea* sp. and mycolic acid-containing bacterium. ***J Antibiot (Tokyo)*** 71(7), 653-657. doi: 10.1038/s41429-018-0040-4.

Hoshino, S., Zhang, L., Awakawa, T., Wakimoto, T., Onaka, H., and Abe, I. (**2015c**). Arcyriaflavin E, a new cytotoxic indolocarbazole alkaloid isolated by combined-culture of mycolic acid-containing bacteria and *Streptomyces cinnamoneus* NBRC 13823. ***J Antibiot (Tokyo)*** 68(5), 342-344. doi: 10.1038/ja.2014.147.

Igarashi, Y., Kim, Y., In, Y., Ishida, T., Kan, Y., Fujita, T., et al. (**2010**). Alchivemycin A, a bioactive polycyclic polyketide with an unprecedented skeleton from *Streptomyces* sp. ***Org Lett*** 12(15), 3402-3405. doi: 10.1021/ol1012982.

Jiang, Y.L., Lu, S., Hirai, G., Kato, T., Onaka, H., and Kakeya, H. (**2019**). Enhancement of saccharothriolide production and discovery of a new metabolite, saccharothriolide C-2, by combined-culture of *Saccharothrix* sp. and *Tsukamurella pulmonis*. ***Tetrahedron Lett*** 60(15), 1072-1074. doi: 10.1016/j.tetlet.2019.03.034.

Jiang, Y.L., Matsumoto, T., Kuranaga, T., Lu, S., Wang, W.C., Onaka, H., et al. (**2021**). Longicatenamides A-D, Two Diastereomeric Pairs of Cyclic Hexapeptides Produced by Combined-culture of *Streptomyces* sp. KUSC_F05 and *Tsukamurella pulmonis* TP-B0596. ***J Antibiot (Tokyo)*** 74(5), 307-316. doi: 10.1038/s41429-020-00400-3.

Kormanec, J., and Sevcikova, B. (2002). The stress-response sigma factor sigma(H) controls the expression of ssgB, a homologue of the sporulation-specific cell division gene ssgA, in Streptomyces coelicolor A3(2). *Mol Genet Genomics* 267(4), 536-543. doi: 10.1007/s00438-002-0687-0.

Latoscha, A., Wormann, M.E., and Tschowri, N. (2019). Nucleotide second messengers in Streptomyces. *Microbiology (Reading)* 165(11), 1153-1165. doi: 10.1099/mic.0.000846.

Liu, X., Zheng, G., Wang, G., Jiang, W., Li, L., and Lu, Y. (2019). Overexpression of the diguanylate cyclase CdgD blocks developmental transitions and antibiotic biosynthesis in Streptomyces coelicolor. *Sci China Life Sci* 62(11), 1492-1505. doi: 10.1007/s11427-019-9549-8.

Lopatniuk, M., Myronovskyi, M., Nottebrock, A., Busche, T., Kalinowski, J., Ostash, B., et al. (2019). Effect of "ribosome engineering" on the transcription level and production of S. albus indigenous secondary metabolites. *Appl Microbiol Biotechnol* 103(17), 7097-7110. doi: 10.1007/s00253-019-10005-y.

Okamoto-Hosoya, Y., Hosaka, T., and Ochi, K. (2003). An aberrant protein synthesis activity is linked with antibiotic overproduction in rpsL mutants of Streptomyces coelicolor A3(2). *Microbiology (Reading)* 149(Pt 11), 3299-3309. doi: 10.1099/mic.0.26490-0.

Oku, N., Takemura, S., Onaka, H., and Igarashi, Y. (**2021**). NMR characterization of streptogramin B and L-156, 587, a non-synergistic pair of the streptogramin family antibiotic complexes produced inductively by a combined culture of *Streptomyces albogriseolus* and *Tsukamurella pulmonis*. ***Magn Reson Chem*** 60(2), 261-270. doi: 10.1002/mrc.5219.

Onaka, H., Ozaki, T., Mori, Y., Izawa, M., Hayashi, S., and Asamizu, S. (**2015**). Mycolic acid-containing bacteria activate heterologous secondary metabolite expression in *Streptomyces lividans*. ***J Antibiot (Tokyo)*** 68(9), 594-597. doi: 10.1038/ja.2015.31.

Ozaki, T., Kurokawa, Y., Hayashi, S., Oku, N., Asamizu, S., Igarashi, Y., et al. (**2016**). Insights into the Biosynthesis of Dehydroalanines in Goadsporin. ***ChemBioChem*** 17(3), 218-223. doi: 10.1002/cbic.201500541.

Ozaki, T., Sugiyama, R., Shimomura, M., Nishimura, S., Asamizu, S., Katsuyama, Y., et al. (**2019**). Identification of the common biosynthetic gene cluster for both antimicrobial streptoaminals and antifungal 5-alkyl-1,2,3,4-tetrahydroquinolines. ***Org Biomol Chem*** 17(9), 2370-2378. doi: 10.1039/c8ob02846j.

Pan, C., Ikeda, H., Minote, M., Tokuda, T., Kuranaga, T., Taniguchi, T., et al. (**2023**). Amoxetamide A, a new anoikis inducer, produced by combined-culture of *Amycolatopsis* sp. and *Tsukamurella pulmonis*. ***J Antibiot (Tokyo)***. doi: 10.1038/s41429-023-00668-1.

Pan, C.Q., Kuranaga, T., Cao, X., Suzuki, T., Dohmae, N., Shinzato, N., et al. (**2021**). Amycolapeptins A and B, Cyclic Nonadepsipeptides Produced by Combined-culture of *Amycolatopsis* sp. and *Tsukamurella pulmonis*. ***J Org Chem*** 86(2), 1843-1849. doi: 10.1021/acs.joc.0c02660.

Rico, S., Santamaria, R.I., Yepes, A., Rodriguez, H., Laing, E., Bucca, G., et al. (2014). Deciphering the regulon of Streptomyces coelicolor AbrC3, a positive response regulator of antibiotic production. *Appl Environ Microbiol* 80(8), 2417-2428. doi: 10.1128/AEM.03378-13.

Sevcikova, B., Benada, O., Kofronova, O., and Kormanec, J. (2001). Stress-response sigma factor sigma(H) is essential for morphological differentiation of Streptomyces coelicolor A3(2). *Arch Microbiol* 177(1)**,** 98-106. doi: 10.1007/s00203-001-0367-1.

Sugiyama, R., Nishimura, S., Ozaki, T., Asamizu, S., Onaka, H., and Kakeya, H. (**2015**). 5-Alkyl-1,2,3,4-tetrahydroquinolines, new membrane-interacting lipophilic metabolites produced by combined culture of *Streptomyces nigrescens* and *Tsukamurella pulmonis*. ***Org Lett*** 17(8)**,** 1918-1921. doi: 10.1021/acs.orglett.5b00607.

Sugiyama, R., Nishimura, S., Ozaki, T., Asamizu, S., Onaka, H., and Kakeya, H. (**2016**). Discovery and Total Synthesis of Streptoaminals: Antimicrobial [5,5]-Spirohemiaminals from the Combined-Culture of *Streptomyces nigrescens* and *Tsukamurella pulmonis*. ***Angew Chem Int Ed Engl*** 55(35)**,** 10278-10282. doi: 10.1002/anie.201604126.

Tschowri, N., Schumacher, M.A., Schlimpert, S., Chinnam, N.B., Findlay, K.C., Brennan, R.G., et al. (2014). Tetrameric c-di-GMP mediates effective transcription factor dimerization to control Streptomyces development. *Cell* 158(5)**,** 1136-1147. doi: 10.1016/j.cell.2014.07.022.

Urem, M., van Rossum, T., Bucca, G., Moolenaar, G.F., Laing, E., Swiatek-Polatynska, M.A., et al. (2016). OsdR of Streptomyces coelicolor and the Dormancy Regulator DevR of Mycobacterium tuberculosis Control Overlapping Regulons. *mSystems* 1(3). doi: 10.1128/mSystems.00014-16.

Willemse, J., Borst, J.W., de Waal, E., Bisseling, T., and van Wezel, G.P. (2011). Positive control of cell division: FtsZ is recruited by SsgB during sporulation of Streptomyces. *Genes Dev* 25(1)**,** 89-99. doi: 10.1101/gad.600211.

Yanagisawa, M., Asamizu, S., Satoh, K., Oono, Y., and Onaka, H. (2022). Effects of carbon ion beam-induced mutagenesis for the screening of RED production-deficient mutants of *Streptomyces coelicolor* JCM4020. *PLoS One* 17(7)**,** e0270379. doi: 10.1371/journal.pone.0270379.
